# Global Trends in Typhoidal Salmonellosis: A Systematic Review

**DOI:** 10.4269/ajtmh.18-0034

**Published:** 2018-07-25

**Authors:** Daina Als, Amruta Radhakrishnan, Paul Arora, Michelle F. Gaffey, Susan Campisi, Russanthy Velummailum, Farhana Zareef, Zulfiqar A. Bhutta

**Affiliations:** 1 Centre for Global Child Health, The Hospital for Sick Children, Toronto, Canada;; 2 Dalla Lana School of Public Health, University of Toronto, Toronto, Canada;; 3 Center of Excellence in Women and Child Health, The Aga Khan University, Karachi, Pakistan

## Abstract

Typhoid and paratyphoid fever continue to significantly contribute to global morbidity and mortality. Disease burden is higher in low-and middle-income settings where surveillance programs are rare and little systematic information exists at population level. This review evaluates national, regional, and global trends in the incidence of typhoid fever and of related morbidity and mortality. A literature search in Medline, Embase, and Web of Science was conducted in June 2016, followed by screening and data extraction in duplicate. Studies reporting blood culture estimates of typhoid or paratyphoid morbidity and mortality were included in the analysis. Our search yielded 5,563 unique records, of which 1978 were assessed for relevance with 219 records meeting the eligibility criteria. *Salmonella enterica* serotype Typhi was the most commonly reported organism (91%), with the occurrence of typhoidal *Salmonella* (either incidence or prevalence) being the most commonly reported outcome (78%), followed by typhoid fever mortality, ileal perforation morbidity, and perforation mortality, respectively. Fewer than 50% of studies stratified outcomes by age or urban/rural locality. Surveillance data were available from 29 countries and patient-focused studies were available from 32 countries. Our review presents a mixed picture with declines reported in many regions and settings but with large gaps in surveillance and published data. Regional trends show generally high incidence rates in South Asia, sub-Saharan Africa, and East Asia and Pacific where the disease is endemic in many countries. Significant increases have been reported in certain countries but should be explored in the context of long-term trends and underlying at-risk populations.

## INTRODUCTION

Typhoid and paratyphoid fever, collectively known as enteric fever, are systemic infections caused by the human-adapted pathogens *Salmonella enterica* serotype Typhi (*S.* Typhi) and *Salmonella enterica* serotype Paratyphi (*S.* Paratyphi) A, B, and C.^1^ Enteric fever continues to be a significant contributor to global morbidity and mortality with an estimated 26.9 million cases and approximately 200,000 deaths directly or indirectly attributable to typhoid each year as of 2010.^1^ According to recent estimates, low-and middle-income countries (LMICs) contribute to almost half of the morbidity and mortality rates of typhoid and paratyphoid fever at 11.9 million cases and 129,000 deaths, respectively.^2^ Similarly, modeled data from the Global Burden of Disease (GBD) Study conducted by the Institute for Health Metrics and Evaluation estimate typhoid and paratyphoid fever morbidity at approximately 15.5 million with 154,000 deaths in 2016.^3^ However, these estimates rely on a modeling approach and given the heterogeneity in terms of diagnostic tools and resource limitations, significant underestimation of disease burden might be occurring. Typhoid and paratyphoid fever can be treated with appropriate antibiotics; however, resistance to first- and second-line antibiotics is a growing public health concern. Effective preventive vaccines exist, and new vaccines are being developed, but corresponding investments in safe drinking water and sanitation infrastructure, food safety, and improved living conditions are insufficient. There are virtually no surveillance programs for typhoid in most LMICs; hence, little systematic information exists on incidence and burden at the population level over time. For a disease as common as enteric fever, such information is essential to guide policy for targeted prevention and control strategies.

**Figure 1.**
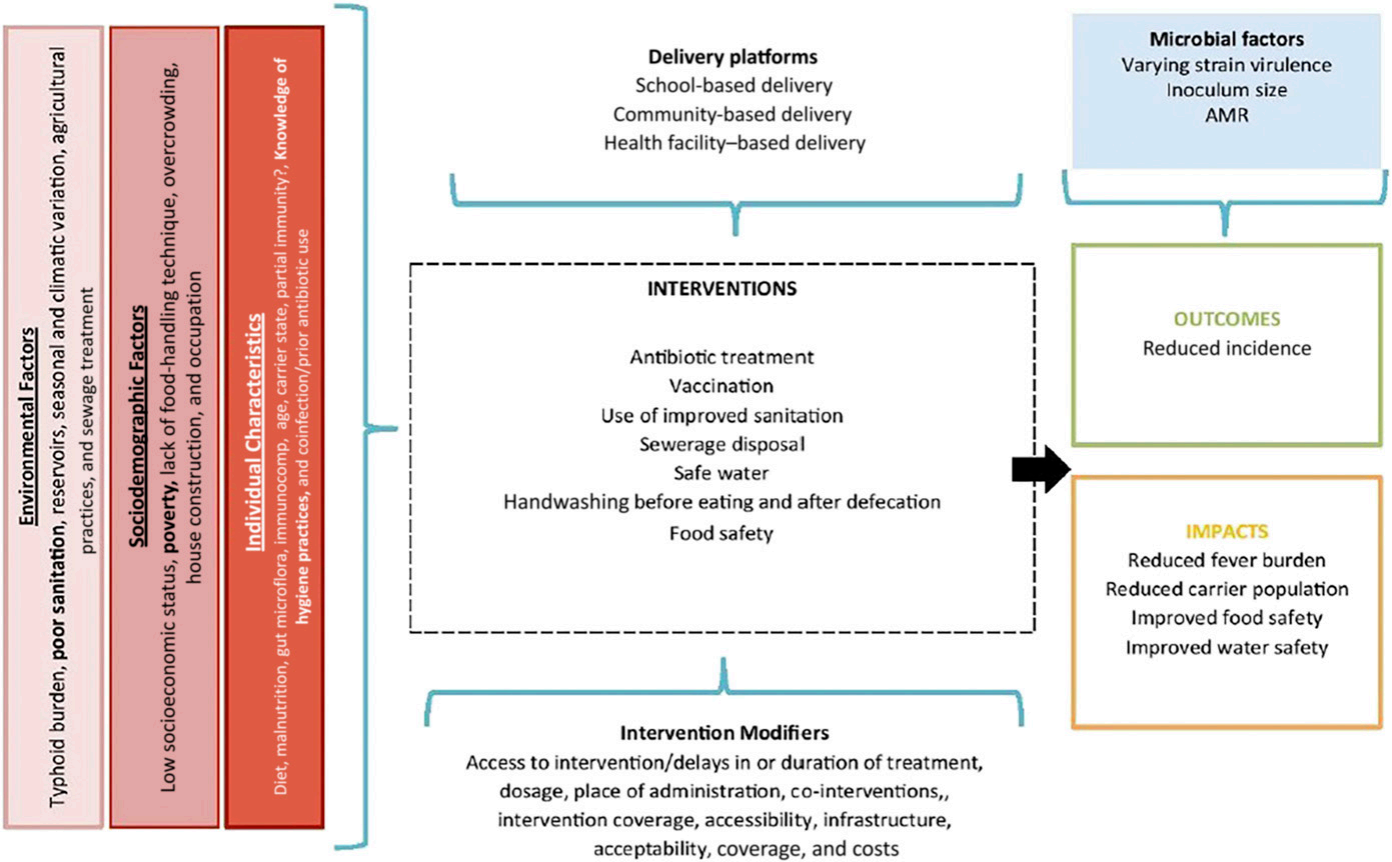
Conceptual framework of typhoid. The conceptual framework describes the environmental and individual-level contextual factors that influence typhoid and paratyphoid fever transmission. It shows the common preventative and curative interventions as well as their delivery platforms and barriers and enablers. It also presents the expected outcomes when control measures are implemented effectively. This figure appears in color at www.ajtmh.org.

Typhoid and paratyphoid fever transmission occurs through ingestion of water or food contaminated with typhoidal *Salmonella* bacteria, with asymptomatic carriers playing an important role in contamination and onward transmission.^4^ Risk factors for typhoid fever include poverty; overcrowding; poor status of water, sanitation, and hygiene (WASH) infrastructure; and poor food-handling practices (Figure 1). Recent estimates suggest that 13% of the global population is living in extreme poverty (defined as people living on less than $1.90/day),^5^ with poverty rates being as high as 60% in some countries,^5^ with deficient WASH infrastructure in many of these settings and some one billion people still practicing open defecation globally.^6^ Despite the persistence of risk factors in many settings, there is a general perception that the burden of typhoid fever has reduced globally, given overall economic growth and development. However, estimates from the 2015 GBD Study suggest that disability-adjusted life years (DALYs) caused by typhoid remain greater than 200 DALYs per 100,000 population in countries across Africa and South Asia, including as many as 587.99 DALYs and 556.88 DALYs per 100,000 population in Burkina Faso and Bangladesh, respectively.^7^ The burden in both countries remains high compared with that in 2010, when 550.24 DALYs were attributable to typhoid in Burkina Faso and 689.91 DALYs in Bangladesh.^7^

Given the continuing importance of typhoid as a public health problem in many settings, we undertook a systematic review of global literature and data to evaluate national, regional, and global trends in the incidence and prevalence of typhoid and paratyphoid fever and related morbidity and mortality in recent decades (1990–2016).

## METHODS

### Search strategy.

To capture all of the relevant peer-reviewed literature on typhoid trends, we applied comprehensive search syntax in multiple electronic databases (Supplemental Appendix 1). The search syntax combined terms relating to typhoid with terms relating to trends, longitudinal data, or surveillance. We searched Medline, Embase, and Web of Science, with no language or date restrictions, in June 2016. We, in addition, searched the gray literature, including conference proceedings, medical textbooks, and government surveillance reports. This was carried out in Google Scholar by modifying the original search syntax. Conference proceedings were obtained from The Coalition against Typhoid for nine international typhoid conferences spanning 1984–2015, and we retrieved relevant chapters on the subject area in standard textbooks of infectious diseases. We searched for government surveillance reports through websites including Ministries of Health, Centers for Disease Control, and Sabin, among others for all countries. To help ensure, we captured all relevant studies and reports, we scanned the reference lists of the 10 most recent relevant systematic review articles, and we contacted experts in typhoid research for any additional items not identified by our other search methods.

### Eligibility.

For a study or report to be eligible for inclusion in our review, it had to 1) report on blood culture–confirmed typhoid or paratyphoid fever; 2) report data from a period of at least 18 months, to account for any seasonality; and 3) report a valid population denominator (i.e., total population or total blood cultures), to allow for quantitative comparisons across time, populations, and settings. The one exception to the aforementioned criteria was the inclusion of government surveillance reports from national disease notification systems which sometimes incorporated other diagnostic methods such as serological testing and stool cultures. These data are presented as crude incidences and were included under the assumption that notification systems would use the reference standard for diagnosis available nationally. Eligible populations included the general population, hospitalized patients, and ambulatory care patients. Studies and reports focusing on a subpopulation with a preexisting morbidity (e.g., human immunodeficiency virus [HIV], asthma, and cancer) were excluded. We also excluded studies or reports focusing on populations in which specific interventions had been evaluated (e.g., vaccines, WASH, or behavior change interventions); however, data from control arms of vaccine trials were included if the study was otherwise eligible. Our main goal was to assess changing incidence and prevalence trends of enteric fever in recent decades in endemic countries. For studies and reports from LMICs, we included only those published from 1990 onward, to assess recent trends. Robust, systematic blood culture data on enteric fever are scarce in developing countries before 1990 because of a lack of resources for surveillance, diagnosis, and treatment; hence, we focused on 1990 onward where data collection was more robust. This cutoff was put in place to capture the most recent culture-confirmed data and the effects of earlier interventions implemented in the 1970s and 1980s that would begin to show their effects in the early 1990s. This time period also aligns with the Millennium Development Goals, allowing for potential comparisons with gains in maternal and child health. This relatively recent time period could also take into account advances in microbiological testing methodologies in these settings. At present, most typhoid and paratyphoid cases in high-income countries (HICs) are imported through travel; we thus applied no date restriction for inclusion of studies from HICs because historical data from these countries are needed to describe longitudinal typhoid trends. Initially, we extracted data on imported cases of enteric fever in HICs, but chose to exclude these studies during the synthesis phase as they do not shed light on disease trends and tend to remain quite low consistently. Finally, we restricted eligibility to studies or reports published or translated into English, as we did not have the capacity to systematically assess non-English texts.

The titles and abstracts of the records identified through our peer-reviewed and gray literature searches were screened for potential eligibility in duplicate; we then screened the full-text publications of those determined to be potentially eligible, also in duplicate. Screening was completed using Covidence software.^8^

### Outcomes.

We included four outcomes in our review: 1) the frequency of blood culture–confirmed *S.* Typhi and *S.* Paratyphi A, B, or C infection; 2) the frequency of typhoid ileal perforation; 3) the frequency of death from typhoid or paratyphoid fever; and 4) the frequency of deaths from typhoid ileal perforation. We extracted and derived several measures of these outcomes for our analyses, described in the following paragraph.

### Data extraction.

Data from eligible peer-reviewed studies were extracted in duplicate and reconciled where necessary through discussion or with input from a third reviewer, whereas data from conference proceedings and government surveillance reports were extracted by a single reviewer. In cases where data were unclear, we attempted to contact the study/report the author for clarification. Where a study spanning more than one year reported data as an aggregate over time, this single data point was assigned to the midpoint year of the study duration. All data, including study/report characteristics and outcome data, were extracted using a standardized extraction form (Supplemental Appendix 2).

### Analysis.

Five main summary measures were derived from the extracted data and used in our analyses, stratified by typhoid and paratyphoid where applicable:

1. Incidence, defined as the number of typhoid or paratyphoid fever cases reported through a notification system per 100,000 people in the general population
2. Prevalence, defined as the number of positive typhoid or paratyphoid cultures as a proportion of the total number of blood samples tested from laboratory surveillance, ambulatory, or hospitalized patients
3. Ileal perforation morbidity, defined as the number of typhoid perforation cases as a proportion of the total number of typhoid cases
4. Case fatality rate, defined as the number of deaths due to typhoid or paratyphoid fever out of the total number of typhoid or paratyphoid cases
5. Perforation fatality rate, defined as the number of deaths from typhoid ileal perforation as a proportion of the total number of typhoid ileal perforations

Annual summary measures were tabulated and plotted, stratified by World Bank regions (East Asia and Pacific, Europe and Central Asia, South Asia, Middle East and North Africa, sub-Saharan Africa, North America, and Latin America and the Caribbean)^9^ and source population type, and 95% confidence intervals (CIs) were calculated for all measures, except the crude incidence rate.

## RESULTS

### Study selection.

**Figure 2.**
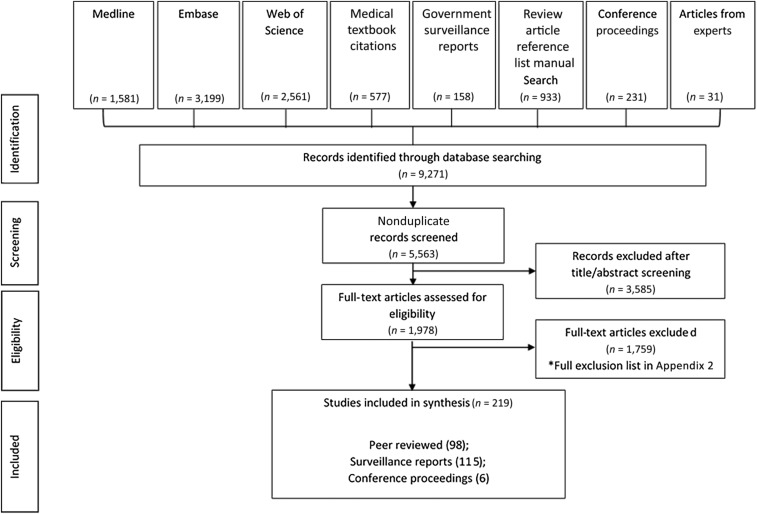
Study flow chart. The preferred reporting items for systematic reviews and meta-analyses flow diagram depicting the movement of records through each stage of the systematic review.

Our search strategy identified 5,563 unique records (Figure 2). Of these, 1978 were assessed as potentially relevant through title and abstract screening, and their full-text reports were retrieved for further assessment of study eligibility. A total of 219 records met the review eligibility criteria, including 98 peer-reviewed studies, 115 government surveillance reports, and six conference proceedings.

### Study characteristics.

Characteristics of the 104 included studies and conference proceedings are shown in Table 1, with the list and citations of the included studies presented in Supplemental Appendix 3. Nearly all studies (91%) reported outcomes for *S.* Typhi, with 30% reporting estimates for *S.* Paratyphi A. Only two (2%) of the included studies reported exclusively paratyphoid outcomes. Outcomes for *S.* Paratyphi B and C were less frequently reported at 8% and 2%, respectively. Although our search strategy and inclusion criteria were broad and inclusive with respect to longitudinal data from all countries, half of the final literature included in our analysis came from only six countries: India, Bangladesh, Pakistan, Nepal, Nigeria, and the United States. National surveillance data were available from only 29 countries, with subnational studies within the general population found from 12 countries, and patient-focused studies (either hospitalized or ambulatory) from 31 countries.

**Table 1:** Study characteristics

		North America	Latin America and Caribbean	Europe and Central Asia	Middle East and North Africa	Sub-Saharan Africa	South Asia	East Asia and Pacific
Outcomes reported	Incidence	0	0	2	3	28	34	11
	Perforation morbidity	0	0	1	1	2	2	1
	Mortality	1	0	1	3	5	4	3
	Perforation mortality	0	0	1	0	4	0	0
Organisms isolated	*S.* Typhi	1	0	3	4	34	36	13
	*S.* Paratyphi A	0	0	0	1	3	22	4
	*S.* Paratyphi B	0	0	1	0	1	5	1
	*S.* Paratyphi C	0	0	0	0	1	1	0
Population type	General population surveillance	3	0	1	1	1	6	4
	Hospitalized patients	1	0	3	3	21	13	6
	Ambulatory patients	0	0	0	0	9	17	7
	Laboratory studies	0	0	0	1	5	10	1

*S*. Paratyphi = *Salmonella enterica* serotype Paratyphi; *S*. Typhi = *Salmonella enterica* serotype Typhi. Table showing metadata for all included peer-reviewed studies identified through the database search.

The frequency of typhoidal *Salmonella* (either incidence or prevalence) was reported in the majority of studies (84%) with typhoid fever mortality, ileal perforation morbidity, and perforation mortality being reported in 17%, 7%, and 5% of studies, respectively. Outcomes were rarely stratified by age and gender. In addition, studies did not commonly report on whether participants were located in urban or rural localities (fewer than 50%), and as a result, no subgroup analyses were possible.

### Typhoid incidence trends in the general population, by region.

#### South Asia.

**Figure 3.**
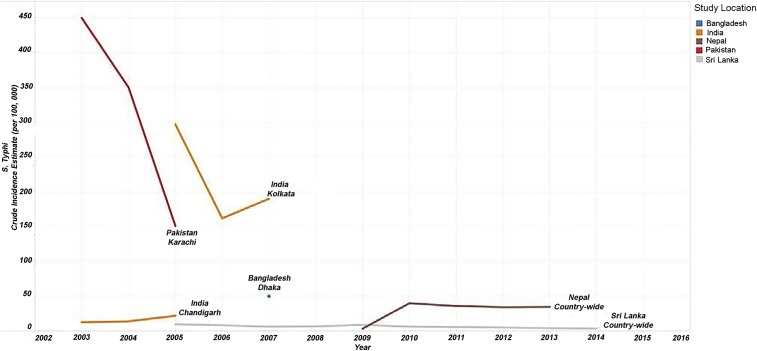
South Asia general population *Salmonella enterica* serotype Typhi (*S.* Typhi) incidence estimates. Line graphs showing the crude incidence estimates per 100,000 per year for countries in South Asia from 2003 to 2014.

We found few government surveillance reports from South Asia that met our eligibility criteria, with longitudinal country-level estimate available only for Nepal and Sri Lanka (Figure 3). Recent data over a 4-year period from Nepal showed a slight decline in typhoid incidence, from 40 cases per 100,000 population in 2010 to 35 cases in 2015; however, incidences have consistently remained within a small range without exhibiting too much variation. Similarly, in Sri Lanka, but where incidence was lower, the number of typhoid cases per 100,000 population decreased from 9.5 in 2005 to 3.5 in 2014. For other South Asian countries, subnational estimates of typhoid incidence in the general population were available from studies in Pakistan, India, and Bangladesh. These trends showed considerable variation ranging from more than 400 cases per 100,000 in 2003 in Karachi, Pakistan, to around 10 cases per 100,000 in Chandigarh, India. However, given the scarcity of time series data from these subnational reasons, these estimates cannot be extrapolated beyond the catchment areas stated in their respective studies.

#### Sub-Saharan Africa.

**Figure 4.**
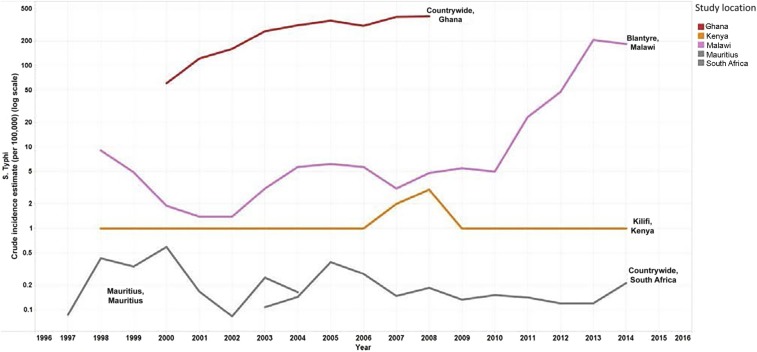
Sub-Saharan Africa general population *Salmonella enterica* serotype Typhi (*S.* Typhi) incidence estimates [log scale]. Line graphs on the logarithmic scale showing the crude incidence estimates per 100,000 per year for countries in sub-Saharan Africa from 1997 to 2014.

National surveillance data from Mauritius and South Africa showed low, relatively stable incidence over time, from 0.1 to 0.6 cases per 100,000 population between 1997 and 2004 in Mauritius, and from 0.1 to 0.3 cases 2003–2014 in South Africa (Figure 4). A steady increase in typhoid incidence was seen over time in Ghana, however, with 60 cases per 100,000 population in 2000 and 400 cases by 2008 based on data obtained from surveillance reports. Data on general population incidence trends in other countries in the region were available from studies in Kilifi, Kenya, and Blantyre, Malawi. Incidence in Kilifi remained relatively constant between 1998 and 2014 at about one case per 100,000 population, with a spike in 2007–2008 to approximately 3.5 cases per 100,000 population. In Blantyre, Malawi, incidence increased sharply between 2010 and 2013, from a relatively stable incidence of about five cases per 100,000 population over the previous decade, to 200 cases per 100,000 population in 2013.

#### East Asia and Pacific.

**Figure 5.**
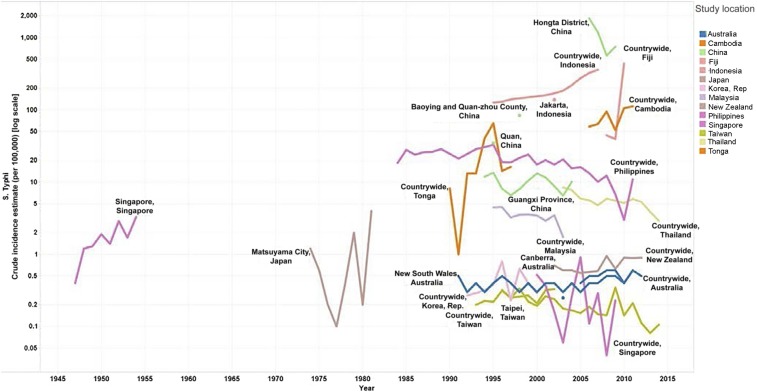
East Asia and Pacific general population *Salmonella enterica* serotype Typhi (*S.* Typhi) incidence estimates [log scale]. Line graphs on the logarithmic scale showing the crude incidence estimates per 100,000 per year for countries in East Asia and the Pacific from 1947 to 2014.

National surveillance data were available from nine countries and additional estimates were available from research studies in four countries, with considerable variability in both the level and trend in typhoid incidence by country. Estimates from HICs such as Australia, Japan, New Zealand, Singapore, South Korea, and Taiwan remained consistently low at less than one case per 100,000 population beginning in 1990 (Figure 5). Data from upper middle-income countries such as China, Fiji, Malaysia, Thailand, and Tonga showed the greatest variation, with Malaysia and Thailand exhibiting declining trends from 4.46 cases per 100,000 in 1995 to 1.74 cases in 2003 and 8.44 in 2003 to 2.92 cases per 100,000 population in 2014, respectively. Conversely, incidences from Fiji consistently remained greater than 40 cases per 100,000, whereas subnational estimates from China ranged from as low as 10 per 100,000 in 2004 in Guangxi Province to 1,850 cases per 100,000 population in 2006 in Hongta district. In lower middle-income countries such as Indonesia, countrywide incidence during the study period increased from 126 cases per 100,000 population in 1995 to 275 in 2005. An increase in typhoid incidence was also observed in Cambodia, from 59 cases per 100,000 in 2006 to 112 in 2011.

#### Middle East and North Africa.

**Figure 6.**
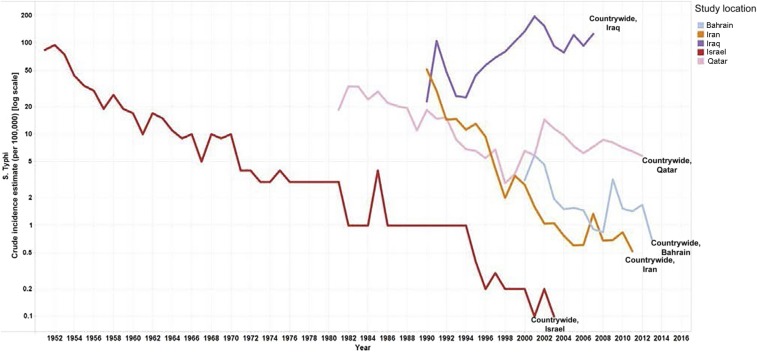
Middle East and North Africa general population *S.* Typhi incidence estimates [log scale]. Line graphs on the logarithmic scale showing the crude incidence estimates per 100,000 per year for countries in Middle East and North Africa from 1951 to 2013.

In this region, national surveillance data from government reports were available for five countries, with Israel, Iran, Bahrain, and Qatar showing similar decreasing trends over time, and Iraq showing a general increase since 1994 and a peak of 196 cases per 100,000 population in 2001 (Figure 6).

**Figure 7.**
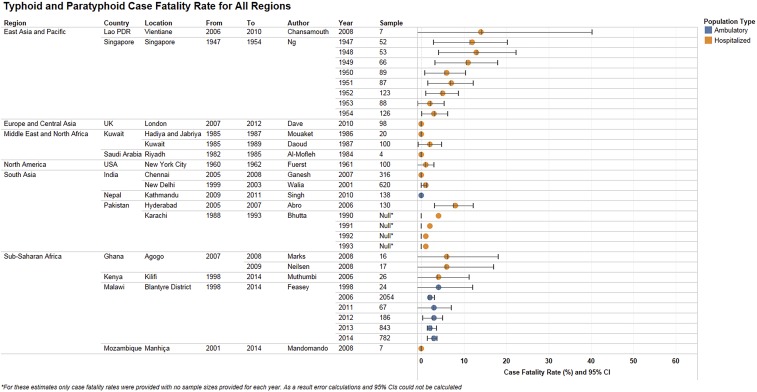
Typhoid and paratyphoid case fatality rates, by region. Case fatality rates due to typhoid and paratyphoid fever as diagnosed by blood culture in hospitalized and ambulatory populations for all regions. Rates are shown as a percentage along with 95% confidence intervals. Null values refer to studies where mortality estimates were provided with no sample size.

#### Latin America and Caribbean.

Government surveillance data were available for Chile from 1975 to 2014 and for Cuba from 1990 to 2015. Data from Chile show a steadily decreasing trend from 121 per 100,000 population in 1978 to 0.4 per 100,000 population in 2014. A series of vaccine trials conducted in schoolchildren in various regions in Santiago, Chile, in the 1980s also showed decreasing typhoid trends in the untreated arms during follow-up. Incidence was consistently low during the surveillance period in Cuba, with a peak of 2.3 cases per 100,000 population in 1993 and no cases since 2007 (Supplemental Appendix 4.1).

#### Europe and Central Asia.

Countrywide incidence data were available over time for only the Netherlands (1995–2006) and Germany (2001–2014), and for a single time point for Finland (2003), with all estimates consistently less than 0.4 cases per 100,000 population. The single available estimate from North Jutland in Denmark in 2003 indicated 0.1 case per 100,000 population, whereas subnational data over time from Madrid in Spain showed a low and consistently declining incidence from the late 1990s to 0.2 cases per 100,000 population by 2005 (Supplemental Appendix 4.2).

#### North America.

Published literature from the United States showed a large decreasing trend from 50 cases per 100,000 population nationally in 1918 to 0.2 per 100,000 population in 1983, mirrored by subnational estimates from New Orleans between 1930 and 1945. Countrywide data from Canada yielded only a single estimate for 2003, at 0.4 cases per 100,000 population (Supplemental Appendix 4.3).

### Paratyphoid incidence trends in the general population.

Compared with typhoid fever, there were limited eligible studies or government surveillance reports that provided data on paratyphoid fever incidence (Supplemental Appendix 4.4). The most common serovar reported was *S.* Paratyphi A, followed by *S.* Paratyphi B and *S.* Paratyphi C. General population incidence of paratyphoid reported in eligible studies and government surveillance reports over time in South Asia and the East Asia and Pacific region showed an overall increasing trend in paratyphoid fever, whereas Europe and Central Asia and Latin America and the Caribbean showed an overall decreasing trend. Regional trends from Middle East and North Africa for 1995–2013 showed a marginally decreasing trend and low rate of paratyphoid fever, with the incidence never climbing higher than 1.3 per 100,000 people in any country. The widest range of incidence rates of paratyphoid among all regions was observed in Latin America and the Caribbean, ranging from 0 to 770 cases per 100,000 spanning from 1983 to 1987. The lowest regional incidence rates were observed in Europe and Central Asia, with all reported incidence rates being lower than 0.1 cases per 100,000 between 2001 and 2014. No incidence data were retrieved from eligible studies or reports for countries in sub-Saharan Africa or North America.

### Trends in typhoid prevalence.

Eligible studies from South Asia included data from Bangladesh, India, Nepal, and Pakistan, with much variability between and within countries (Supplemental Appendix 5.1). There were insufficient data from the region as a whole. This, paired with the heterogeneity in the time periods, patient characteristics and study locations meant that no pooled regional or national estimates could be calculated. The trend data presented here show generally higher prevalence estimates (ranging from 4% to 37%) in the hospitalized patient population compared with the ambulatory or laboratory surveillance groups, although this can partially be explained by the small sample sizes in this study. The highest prevalences and the largest degree of variability were generally present in the included studies from India followed by Nepal.

In sub-Saharan Africa, typhoid data came mostly from hospitalized patients, followed by ambulatory patients and laboratory isolates, and covered 14 countries (Supplemental Appendix 5.2). Countrywide estimates were available from Uganda (1996–2007), Kenya (2006–2009), the Democratic Republic of the Congo (2007–2011), and Ghana (2010–2013). Outside of Nigeria, the prevalence of blood culture–proven *S.* Typhi remained less than 10%. Time series data, which are generally rare, from Blantyre in Malawi showed prevalence rates that remained close to 1% between 1998 and 2010 before starting to exhibit an upward trend in the following years, leading to a prevalence of 5.72% (95% CI: 5.33, 6.11) in 2013.

Most of the eligible studies from East Asia and Pacific analyzed samples from hospitalized populations, followed by ambulatory patients and laboratory studies. The eligible studies reported wide variation in *S.* Typhi positivity rates between countries and over time, ranging from 0.05% among ambulatory patients in Thailand in 2007 to 24.1% among ambulatory patients in Cambodia in 2009. Data were available from six countries in this region, including countrywide estimates from Hong Kong and Thailand (Supplemental Appendix 5.3). Subnational estimates showed considerable variation with the trend from Tagbilaran City in the Philippines, showing a generally decreasing trend from 1994 to 1997, whereas subnational data from Songkhla, Thailand, rose from 4% to 10.2% in 2011 within a year due to an outbreak.

Studies from the Middle East and North Africa reported prevalence rates mostly from laboratory studies. Serial data from one study in Kuwait covering 1995–2002 reported relatively low rates of typhoid over time, with the percent positivity remaining less than 4% (Supplemental Appendix 5.4). Additional aggregate estimates from Saudi Arabia in the early 1980s reported a prevalence of 6.5% (95% CI: 0.3, 12.6).

Estimates from Europe and Central Asia were available from Georgia and Turkey only from hospitalized patients (Supplemental Appendix 5.5). Given that one of the two available estimates (Turkey) was from a pediatric population, no meaningful trend could be discerned for this region.

### Trends in paratyphoid prevalence.

The organism most commonly reported on was *S.* Paratyphi A, followed by B and C. Eligible studies from three countries within South Asia presented paratyphoid data and were included in this review (Supplemental Appendix 5.6). Eligible studies from India reported primarily on *S*. Paratyphi A, mostly from subnational studies of laboratory specimens but also among ambulatory and hospitalized patients. Estimates of *S.* Paratyphi A prevalence were generally less than 8% across study sites and populations and over time, except in Coimbatore (32% among ambulatory patients in 2004). Only two studies reported on *S.* Paratyphi B, from Coimbatore (1.05% in 2003 and 1.75% in 2004) and Delhi (0.01% for the period 2010–2012), both among ambulatory patients. The longest time series from the region spans 1993–2003 in a study from Kathmandu, Nepal, and across all six included studies from Kathmandu, *S.* Paratyphi A prevalence among ambulatory patients ranged from less than 1% in 2003 to 8% in 2010. Five studies from Pakistan reported on *S.* Paratyphi A prevalence, with three of these also reporting on *S.* Paratyphi B. Prevalence rates for both remained less than 4% across study sites and over time, with the exception of one study in Karachi that reported an *S.* Paratyphi A rate of 15% for the period 2008–2010.

In subnational studies from Burkina Faso, Ghana, Guinea-Bissau, Kenya, Madagascar, Mozambique, Tanzania, and Senegal, *Salmonella* Paratyphi A, B, and C prevalence rates were estimated at less than 0.7% over each study period.

In East Asia and Pacific countries, data on paratyphoid were available from studies on hospitalized patients, ambulatory patients, or laboratory specimens from Cambodia, Hong Kong, and the Philippines. Data representative of the whole of Hong Kong were available for the period 1984–1989 for *S.* Paratyphi A and B, with prevalence estimated at 0.3% and 4.9%, respectively. *S.* Paratyphi A positivity was estimated at less than 1.9% among study populations in Cambodia and the Philippines over each study period.

Finally, only one study from the Middle East and North Africa region that met the review inclusion criteria reported on paratyphoid rates. In Kuwait, *S.* Paratyphi A prevalence based on laboratory specimens ranged from 0.11% to 0.82% across the period 1995–2002.

### Secondary outcomes.

#### Trends in typhoid ileal perforation rates*.*

Typhoid ileal perforation could not be segregated by region because of the small number of studies (*N* = 7) reporting this outcome (Supplemental Appendix 6.1). The ileal perforation rate ranged from 0% to 60%; however, discounting outliers, most of the studies reported perforation rates between 0% and 4%.

#### Trends in typhoid and paratyphoid death rates.

Case fatality rates varied considerably across geography, time, and organism within the studies included in this review, with 0% case fatality being the most commonly reported estimate, and the highest being 14.3% fatality among a small number of cases from a study in Vientiane, Laos, between 2006 and 2010 (Supplemental Appendix 6.2). Countrywide historical mortality data were only available from Singapore, where the case fatality rate showed a decreasing trend from 1947 to 1954. Studies that reported typhoid perforation–related deaths had small sample sizes ranging from 14 to 75 (Supplemental Appendix 6.3). Studies with relatively large sample sizes (greater than 50) reported perforation case fatality rates between 20% and 34%, with very large CIs.

## DISCUSSION

### Summary of results.

Our review of trends in culture-proven typhoidal *Salmonella* from the global literature presents a mixed picture with broad declines reported in many regions and settings but with large gaps in surveillance and published data. For example, despite the fact that most countries in sub-Saharan Africa are endemic for typhoid fever, the prevalence of this disease in the region is poorly defined in existing literature and could reflect the paucity of robust reporting systems and population-based research in this area. These gaps highlight the need for standard notification systems across countries to better characterize the global burden of typhoid and paratyphoid fever. Regional trends show generally high incidence rates in South Asia, sub-Saharan Africa, and East Asia and Pacific where typhoid and paratyphoid fever are endemic diseases in many countries. Regions such as North America and Europe and Central Asia reported higher rates of typhoid and paratyphoid fever historically with a decreasing trend over time. Presently, most of the newly diagnosed cases of typhoid and paratyphoid fever in these regions are imported through travel from endemic areas/populations. Most of the included literature reported data on typhoid fever outcomes with paratyphoid fever being relatively underrepresented. This is particularly interesting, given the inverse trends observed in certain regions with paratyphoid fever incidence increasing over time as typhoid fever incidence decreased.

Significant increases have been reported in certain countries but should be explored in the context of long-term trends and underlying at-risk populations. For example, surveillance data from Cambodia (2005–2011) report increases in typhoid and paratyphoid fever cases with published data from ambulatory populations, suggesting a higher prevalence of typhoid than in neighboring countries.^10–15^ On the other hand, surveillance from Indonesia (from the mid-1990s to the mid-2000s) also depicted an increasing trend in contrast to the data from peer-reviewed studies that showed a relatively low prevalence of typhoid and paratyphoid fever in ambulatory patients.^16^ Our review highlights the large variability in typhoid and paratyphoid fever incidence and prevalence data, and while regional summation is reasonable, there is insufficient high-quality data to comment on national and subnational trends.

Prevalence of comorbidities such as HIV, differences in antimicrobial resistance patterns, over-the-counter antibiotic availability, substandard antibiotic preparations, lack of pipe-borne potable water, health system functionality, and health-seeking behaviors all weigh into the differences seen in disease spectrum, complications, and mortality across regions.^17^ In addition, reports suggest considerable influence of age, with some studies documenting increased morbidity and mortality in younger children,^18–21^ whereas others report comparatively better outcomes in this age group. The emergence of and subsequent increasing trend in single- and multidrug-resistant typhoidal *Salmonella*^18,19^ have been associated with reported changes in disease severity.^22–24^ This has narrowed the therapeutic options in high-burden countries and has also led to increased treatment costs, severity of illness, and higher case fatality rates.^22,25–28^

### Limitations and gaps in knowledge.

Our review was limited by the availability and quality of data in the published literature. Although we included estimates that fit our inclusion criteria where possible, meaningful trends or clustering was not readily evident. The main reason for this is the heterogeneity in the reporting of typhoid and paratyphoid fever research. There was a notable amount of variation in testing methodologies in the published included literature. Presently, blood cultures are the gold standard for diagnosing typhoid, but only 40% of extracted studies reported estimates based on this test method and were used for our analysis. In addition, data from government reports were included regardless of the diagnostic method used. This exception was made because these reports tended to be nationally representative and would provide a more accurate picture of population-level disease burden as opposed to estimates that only apply to the catchment areas of hospitals and clinics.

Inconsistency in testing methodology and limited studies reporting time series data rendered robust statistical analysis difficult. Laboratory samples were not always restricted to one sample per patient and data on blood culture volumes were not available. Thus, the blood culture–proven figures and rates could well represent underestimates due to poor sensitivity of small volume of blood cultures and varying methodologies.^29^ In addition, restricting the results to only include blood culture–confirmed estimates introduces selection bias because only institutions where the resources necessary to perform sophisticated laboratory tests were included. Given that typhoid fever more commonly occurs in resource limited settings, this inclusion criterion could have led to further underestimation of disease burden. The diversity encountered in incidence estimates due to the use of nonstandardized test methods, as well as the poor sensitivity of blood cultures highlights the need for establishing a common test methodology for global typhoid and paratyphoid fever estimates.

Roughly, half of the published data on typhoidal *Salmonella* did not present age-stratified data. This makes it difficult to explore reports that school-aged children are at much higher risk of infection with typhoidal *Salmonella* than children less than 5 years of age.^29^ Furthermore, the importance of age-stratified trends is highlighted by evidence that the average age of typhoid infection is lower in areas of higher incidence.^30^ Included studies generally reported incidence data as diagnosed by local hospitals, laboratories, and institutions, and hence may not be nationally representative. Furthermore, subnational hospital/clinic-based estimates generally only apply to very specific populations living under very fixed conditions; hence, these results cannot be extrapolated to the country or provincial/state level in most cases. This further strengthened the basis for undertaking more robust national case studies using best possible laboratory sources of culture-confirmed typhoid cases.

It is important to note that this review could have been affected by publication bias. Certain authors published multiple articles at different times that reported on the same population (e.g., vaccine trial follow-ups). In such cases, the studies were assessed for quality, and only estimates from the study reporting more complete and recent data were kept. Because of logistical constraints, we were unable to undertake full-text screening and data extraction from non-English studies that were otherwise eligible. This may have limited our results from China and Taiwan, where a large body of medical literature exists in Mandarin. From the initial set of studies that were determined to be relevant for this review, close to 60% had to be excluded for a variety of reasons (nonblood culture–confirmed diagnoses, data on special populations, etc.) As a result, the estimates presented here might not reflect the true burden of disease in the included settings. Only 14 studies reported on data collected at the same location over multiple consecutive years. These studies varied in terms of the type of population (hospitalized patients, outpatients, or laboratory samples) and were often very specific to a clinic or hospital. For this reason, we chose to not report on such studies separately, although our initial aim was to present them as a separate analysis. Furthermore, certain countries (such as India, Nigeria, and the United States) represent a sizeable portion of the included studies. As a result, an estimate of the true GBD could not be presented, given the dearth of systematic data from most endemic countries.

## CONCLUSION

Our systematic review suggests that although typhoid incidence has decreased in most developed countries, it remains prevalent in many LMICs. There was reduction in the rates of typhoid with evidence of rates having stabilized to low levels of endemicity in some countries and virtual disappearance of the disorder in others as a public health problem such as in Vietnam and Thailand. To assess in depth the status on trends and determinants, further country-based analysis using mixed methods might provide greater insight into trends of typhoid and factors associated with change.

The diagnosis of typhoid and paratyphoid fever can often be under- or overestimated depending on the test methods, highlighting the need for the establishment of an accurate standard diagnosis tool. At present, no vaccines exist for paratyphoid fever which likely plays an important role in the relative increase in the proportion of these isolates as rates of typhoid fever have reduced. Further research is needed to understand the risk factors and epidemiology of paratyphoid fever and its control.

Our report provides a comprehensive picture of the burden of typhoid and paratyphoid fever around the world. We aimed to be inclusive with data collection and chose to present our results as a narrative as opposed to a meta-analysis to highlight the variability in typhoid and paratyphoid fever reporting. This review can be a guiding tool for policy-makers and academics as it summarizes the current state of typhoid research and highlights important knowledge gaps that need to be explored. Further mathematical modeling of clinical and surveillance data on typhoidal *Salmonella* should be pursued.

## Supplementary Material

Supplemental appendices
