# Supplementary material for: Global Trends in Typhoidal Salmonellosis: A Systematic Review
Source: Am J Trop Med Hyg. 2018 Jul 25;99(3 Suppl):10–9. doi: 10.4269/ajtmh.18-0034 (PMC6128363; doi:10.4269/ajtmh.18-0034)
Supplement: Supplementary file 1 [file tpmd180034.SD1.pdf]

Appendix 1: Search Syntax

Appendix 1.1 Medline

1. **Enteric Fever Terms**
   exp Typhoid Fever/ OR exp Salmonella Typhi/ [MeSH] OR typhoid fever.ti,ab.OR Enteric fever.ti,ab.OR exp Paratyphoid fever/ [MeSH] OR exp Salmonella Paratyphi A/ [MeSH] OR exp Salmonella Paratyphi B/ [MeSH] OR exp Salmonella Paratyphi C/ [MeSH] OR Paratyphoid fever.ti,ab.
2. **Case Countries**
   Chile.mp. OR exp Chile/ OR Egypt.mp. OR exp Egypt/ OR South Africa.mp. OR exp South Africa/ OR Thailand.mp. OR exp Thailand/ OR Vietnam.mp. OR exp Vietnam/ OR India.mp. OR exp India/ OR Pakistan.mp. OR exp Pakistan/
3. **Longitudinal Terms**
   incidence.ab,ti. OR rate$.ab,ti. OR frequency.ab,ti. OR prevalence.ab,ti. OR morbidity.ab,ti. OR burden.ab,ti. OR surveillance.ab,ti. OR epidemiology.ab,ti. OR trend$.ab,ti. OR exp Time Factors/ [MeSH] OR Time series.mp. OR longitudinal.mp. OR cohort.ab,ti. OR mortality.ab,ti.
4. **Developed Countries**
   Norway.mp. OR exp Norway/ OR Australia.mp. OR exp Australia/ OR Switzerland.mp. OR exp Switzerland/ OR Netherlands.mp. OR exp Netherlands/ OR United States.mp. OR exp United States/ OR Germany.mp. OR exp Germany/ OR New Zealand.mp. OR exp New Zealand/ OR Canada.mp. OR exp Canada/ OR Singapore.mp. OR exp Singapore/ OR Denmark.mp. OR exp Denmark/ OR Ireland.mp. OR exp Ireland/ OR Sweden.mp. OR exp Sweden/ OR Iceland.mp. OR exp Iceland/ OR United Kingdom.mp. OR exp United Kingdom/ OR Hong Kong,China.mp. OR exp Hong Kong,China/ Hong Kong, China (SAR).mp. OR exp Hong Kong, China(SAR)/ OR Republic of Korea.mp. OR exp Republic of Korea/ OR Japan.mp. OR exp Japan/ OR Liechtenstein.mp. OR exp Liechtenstein/ OR Israel.mp. OR exp Israel/ OR France.mp. OR exp France/ OR Austria.mp. OR exp Austria/ OR Belgium.mp. OR exp Belgium/ OR Luxembourg.mp. OR exp Luxembourg/ OR Finland.mp. OR exp Finland/ OR Slovenia.mp. OR exp Slovenia/ OR Italy.mp. OR exp Italy/ OR Spain.mp. OR exp Spain/ OR Czech Republic.mp. OR exp Czech Republic/ OR Greece.mp. OR exp Greece/ OR Brunei Darussalam.mp. OR exp Brunei Darussalam/ OR Qatar.mp. OR exp Qatar/ OR Cyprus.mp. OR exp Cyprus/ OR Estonia.mp. OR exp Estonia/ OR Saudi Arabia.mp. OR exp Saudi Arabia/ OR Lithuania.mp. OR exp Lithuania/ OR Poland.mp. OR exp Poland/ OR Andorra.mp. OR exp Andorra/ OR Slovakia.mp. OR exp Slovakia/ OR Malta.mp. OR exp Malta/ OR United Arab Emirates.mp. OR exp United Arab Emirates/ OR Chile.mp. OR exp Chile/ OR Portugal.mp. OR exp Portugal/ OR Hungary.mp. OR exp Hungary/ OR Bahrain.mp. OR exp Bahrain/ OR Cuba.mp. OR exp Cuba/ OR Kuwait.mp. OR exp Kuwait/ OR Croatia.mp. OR exp Croatia/ OR Latvia.mp. OR exp Latvia/ OR Argentina.mp. OR exp Argentina/
5. **Combinations**
   1 and 3
   1 and 2 and 3
   1 and 3 and 4

Appendix 1.2 Embase

1. **Enteric Fever Terms**
   (typhoid fever$ or typhoid or typhus abdominal or fevers enteric or fever enteric or abdominal typhus or enteric fever$ or fever typhoid or typhoids).mp. or (salmonella typhi or salmonella typhosa or salmonella enterica serovar typhi or "S. Typhi").mp. Or Paratyphoid fever.mp. or Paratyphoid fever.fs.or Salmonella Paratyphi A.mp. or Salmonella Paratyphi A.fs.or Salmonella Paratyphi B.mp. or Salmonella Paratyphi B.fs.OR Salmonella Paratyphi C.mp. OR Salmonella Paratyphi C.fs.
2. **Case Countries**
   Chile.mp. OR Chile.fs. OR Egypt.mp. OR Egypt.fs. OR South Africa.mp. OR South Africa.fs. OR Thailand.mp. OR Thailand.fs. OR Vietnam.mp. OR Vietnam.fs. OR India.mp. OR India.fs. OR Pakistan.mp. OR Pakistan.fs.
3. **Longitudinal Terms**
   incidence.ab,ti.OR rate$.ab,ti. OR Frequency.ab,ti. OR prevalence.ab,ti.OR Morbidity.ab,ti. OR burden.ab,ti. OR surveillance.ab,ti. OR epidemiology.ab,ti. OR trend$.ab,ti.OR Time Factors.mp. OR Time series.mp. OR longitudinal.mp. OR cohort.ab,ti. OR mortality.ab,ti.
4. **Developed Countries**
   Norway.mp. OR Norway.fs. OR Australia.mp. OR Australia.fs. OR Switzerland.mp. OR Switzerland.fs. OR Netherlands.mp. OR Netherlands.fs. OR United States.mp. OR United States.fs. OR Germany.mp. OR Germany.fs. OR New Zealand.mp. OR New Zealand.fs. OR Canada.mp. OR Canada.fs. OR Singapore.mp. OR Singapore.fs. OR Denmark.mp. OR Denmark.fs. OR Ireland.mp. or Ireland.fs. OR Sweden.mp. OR Sweden.fs. OR Iceland.mp. or Iceland.fs. OR United Kingdom.mp. OR United Kingdom.fs. OR (Hong Kong,China OR Hong Kong, China SAR).mp. OR (Hong Kong,China OR Hong Kong, China SAR).fs. OR Republic of Korea.mp. OR Republic of Korea.fs. OR Japan.mp. OR Japan.fs. OR Liechtenstein.mp. OR Liechtenstein.fs. OR Israel.mp. OR Israel.fs. OR France.mp. OR France.fs. OR Austria.mp. OR Austria.fs. OR Belgium.mp. OR Belgium.fs. OR Luxembourg.mp. OR Luxembourg.fs. OR Finland.mp. OR Finland.fs. Slovenia.mp. OR Slovenia.fs OR Italy.mp. OR Italy.fs. OR Spain.mp. OR Spain.fs. OR Czech Republic.mp. OR Czech Republic.fs. OR Greece.mp. OR Greece.fs. OR Brunei Darussalam.mp. OR Brunei Darussalam.fs. OR Qatar.mp. OR Qatar.fs. OR Cyprus.mp. OR Cyprus.fs. OR Estonia.mp. OR Estonia.fs. OR Saudi Arabia.mp. OR Saudi Arabia.fs. OR Lithuania.mp. OR Lithuania.fs. OR Poland.mp. OR Poland.fs. OR Andorra.mp. OR Andorra.fs. OR Slovakia.mp. OR Slovakia.fs.OR Malta.mp. OR Malta.fs. OR United Arab Emirates.mp. OR United Arab Emirates.fs. OR Chile.mp. OR Chile.fs. OR Portugal.mp. OR Portugal.fs.OR Hungary.mp. OR Hungary.fs. OR Bahrain.mp.OR Bahrain.fs.OR Cuba.mp. OR Cuba.fs. OR Kuwait.mp. or Kuwait.fs.OR Croatia.mp. OR Croatia.fs. OR Latvia.mp. OR Latvia.fs. OR Argentina.mp. OR Argentina.fs.
5. **Combinations**
   1 and 3
   1 and 2 and 3
   1 and 3 and 4

Appendix 1.3 Web of Science

1. **Enteric Fever Terms**
   TS=(typhoid fever$ OR typhoid OR typhus abdominal OR fevers enteric OR fever enteric OR abdominal typhus OR enteric fever$ OR fever typhoid OR typhoids OR salmonella typhi OR salmonella typhosa OR salmonella enterica serovar typhi OR "S. Typhi" OR Paratyphoid fever OR Salmonella Paratyphi A OR Salmonella Paratyphi B OR Salmonella Paratyphi C)
2. **Case Countries**
   CU=Chile OR Egypt OR "South Africa" OR Thailand OR Vietnam OR India OR Pakistan)
   Indexes=SCI-EXPANDED, SSCI, A&HCI, CPCI-S, CPCI-SSH Timespan=1990-2016
3. **Longitudinal Terms**
   TS=incidence*OR rate$ OR frequency OR prevalence$ OR morbidit* OR burden OR surveillance OR epidemiology OR trend$ OR Time Factor$ OR Time series OR longitudinal OR cohort OR studies incidence OR cohort study OR cohort studies historical OR cohort studies closed OR cohort studies OR closed cohort study OR historical cohort study OR studies historical cohort OR study incidence OR cohort study closed OR study cohort OR cohort analyses OR studies cohort OR analysis cohort OR analyses cohort OR historical cohort studies OR incidence study OR study closed cohort OR study historical cohort OR studies closed cohort OR incidence studies OR studies concurrent OR cohort study historical OR closed cohort studies OR study concurrent OR concurrent study OR concurrent studies OR cohort analysis OR mortality OR decline mortality OR rates age-specific death OR mortality excess OR mortality determinant OR rate death OR rate mortality OR differential mortality OR death rates age-specific OR differential mortalities OR rate age-specific death OR rates death OR death rates OR age-specific death rate OR excess mortality OR determinants mortality OR mortality decline OR mortality rates OR death rate age-specific OR age-specific death rates OR mortalities differential OR case fatality rate OR case fatality rates OR mortalities OR mortality OR determinant mortality OR rates case fatality OR mortality declines OR mortality rate OR declines mortality OR excess mortalities OR rates mortality OR rate case fatality OR age specific death rate OR death rate OR mortality determinants OR mortality differential OR mortalities excess)
4. **Developed Countries**
   CU=(Norway OR Australia OR Switzerland OR Netherlands OR USA OR Germany OR New Zealand OR Canada OR Singapore OR Denmark OR Ireland OR Sweden OR Iceland OR United Kingdom OR Hong Kong OR China OR Korea OR Republic of Korea OR Japan OR Liechtenstein OR Israel OR France OR Austria OR Belgium OR Luxembourg OR Finland OR Slovenia OR Italy OR Spain OR Czech Republic OR Greece OR Brunei Darussalam OR Brunei OR Qatar OR Cyprus OR Estonia OR Saudi Arabia OR Lithuania OR Poland OR Andorra OR Slovakia OR Malta OR United Arab Emirates OR Chile OR Portugal OR Hungary OR Bahrain OR Cuba OR Kuwait OR Croatia OR Latvia OR Argentina
5. **Combinations**
   1 and 3
   1 and 2 and 3
   1 and 3 and 4

Appendix 1.4
Google Books
Enteric Fever and Longitudinal Terms
"Typhoid Trends", "Typhoid Rate", "Typhoid Incidence" and "Typhoid Prevalence"

Appendix 1.5
PubMed Bookshelf

1. **Enteric Fever Terms**
   Typhoid Fever OR Salmonella Typhi OR Enteric fever OR Paratyphoid fever OR Salmonella Paratyphi OR Salmonella Paratyphi A OR Salmonella Paratyphi B OR Salmonella Paratyphi C
2. **Longitudinal Terms**
   incidence OR rate OR prevalence OR mortality OR burden OR trend* OR morbidity OR longitudinal OR surveillance OR cohort OR histor*
3. **Combinations**
   1 and 2

Appendix 1.6
Review Articles; Medline

1. Enteric Fever Terms
   Typhoid Fever OR Salmonella Typhi OR Enteric fever OR Paratyphoid fever OR Salmonella Paratyphi OR Salmonella Paratyphi A OR Salmonella Paratyphi B OR Salmonella Paratyphi C
2. **Longitudinal Terms**
   incidence OR rate OR prevalence OR mortality OR burden OR trend* OR histor* OR longitudinal OR surveillance OR morbidity OR cohort
3. **Combinations**
   1 and 2
