# Supplementary material for: Global Trends in Typhoidal Salmonellosis: A Systematic Review
Source: Am J Trop Med Hyg. 2018 Jul 25;99(3 Suppl):10–9. doi: 10.4269/ajtmh.18-0034 (PMC6128363; doi:10.4269/ajtmh.18-0034)
Supplement: Supplementary file 2 [file tpmd180034.SD2.pdf]

Appendix 2: Data Dictionary and Full-Text Exclusion Reasons

Appendix 2.1: Data Dictionary

	Definition and instructions	Data validation	example values	Validation values																										
**Study Report Identifiers and Characteristics**																														
Study ID#	Serial number to identify a study uniquely - please copy exactly as appears on Data Extraction Division of Work Sheet	n	TT775; TT748..TT####																											
First Author Last Name	Last name of the first author of the publication																													
Publication Year	Year article was published																													
Article Title	Title of publication																													
Journal name	Journal of Publication																													
Contact Author?	Need to contact author(s) for additional data from the study or to ask for clarification?	y	yes, no	y	n																									
Incidence/prevalence	Incidence/prevalence rate reported?	y	yes, no	y	n																									
Morbidity	Typhoid ileal perforation rate reported?	y	yes, no	y	n																									
Mortality	Death rate reported?	y	yes, no	y	n																									
Perforation Mortality	Deaths caused by typhoid ileal perforation rate reported?	y	yes, no	y	n																									
**Study Population Characteristics**																														
Geographic Region (according to W orld Bank)	Regions of the W orld as defined by the World Bank (Sub-Saharan Africa, Latin America and Caribbean, East Asia and Pacific, Europe and Central Asia, South Asia, Middle East and North Africa, North America) (http://data.worldbank.org/about/country-and-lending-groups)	y	Drop-down menu																											
Country	Country name; If traveller study, country where study is conducted																													
Location	Specifiy as much detail as possible about the study location (i.e. Province, town, village, hospital, etc.)																													
Human Development Index Ranking (UN)	As defined by United Nations (http://hdr.undp.org/en/content/table-1-human-development-index-and-its-components)	y	Drop-down menu																											
Income (according to World Bank)	Income categories as defined by the World Bank (Low income, lower middle income, upper middle income, high income) (http://data.worldbank.org/about/country-and-lending-groups#Lower_middle_income)	y	1=yes, 0=no																											
Currently Endemic	Countries listed as endemic as defined by the IAMAT (https://www.iamat.org/risks/typhoid-fever)	y	yes, no	y	n																									
Study Sample Size	Number of participants included in the study overall, unstratified, that fit the study selection criteria - included details in Comments section																													
Gender	Male, Female or both	y	Drop-down menu	Male	Female	Both																								
Female (%)	Percentage of females in overall study population		25%, 30%																											
Age of Sample	Any age; can include age range if a point estimate of age is not reported. Be sure to include the units and the type of measurement (range, average, mean, median)		34, 15-49, 6 months																											
Urban/Rural	Urban or rural population as reported by the study. If not reported, look up the description of the study location and asses urban or rural. Record as completely as possible the geographic information reported by the study.	y	u=urban, p=periurban, r=r	u	p	r																								
Proportion of population immunized	Immunization rate within study population (as reported by study)		5%, 25%																											
Outbreak?	Does the study present data from an outbreak (as defined by the study)?		y, n, u																											
Traveller Study?	Does the study include outcomes of interest pertaining to travellers?		y, n																											
Study Comments	Add any comments pertaining to study that are not included in other columns (e.g. Severity of Typhoid was assessed)																													
Study Design	Cohort, cross-sectional, longitudinal, etc. (E.g. if same participants are not followed over time, then cross-sectional); Contact research supervisors if unsure.		pull down menu	Cross- sectional	or Repeated cross sectional	Cohort	Case-control	RCT	Quasi-experimental	Other/unsure																				
Years of Case Ascertainment	Year of start and end of study; Any range of years		1994, 2003																											
Multi-year Single Data Point	Does study report single point prevalence for a range of years? In this case, data for multiple years are groups/aggregated together as a single		yes, no																											
Sources of Data	Single- or multi-centre study location.	y	single, multicentre, not reported	single	multicentre	not reported																								
Method of Diagnosis	Tests that were used for identification and/or confirmation of serovar including Bone marrow, blood culture, stool culture, PCR, Widal test, serology, or others	y	1=yes, 0=no, -=at least one serovar identification/confirmation test performed, but study does not specify	1	0	-																								
Bacterium Serovar	Five options: S. Typhi, S. Paratyphi A, S. Paratyphi B or S. Paratyphi C, multiple	y	1=yes, 0=no	1	0																									
Inclusion of Suspect Cases	Skip. If they have lab verification of infection	y	1=yes, 0=no	1	0																									
Antimicrobial resistance test	Disk diffusion, PCR, DNA hybridization, E-test, microbroth dilution, agar dilution, or others	y	1=yes; 0=no (not done); - = at least one AMR test performed, but study does not specify	1	0	-																								
**TrendsOutcomes**																														
Study Location	State and Country information; especially important for studies that stratify by location; If traveller study, put country where study is conducted																													
Organism	S. Typhi, S. Paratyphi A, S. Paratyphi B or S. Paratyphi C; Assume S. Typhi for "enteric fever"; Assume S. Paratyphi A for "paratyphoid fever"	y	pull down menu																											
Time: Month and Year	Midpoint time (month/year) estimate of rate (incidence, prevalence, mobidity, mortality)	y	jan	feb	mar	apr	may	jun	jul	aug	sep	oct	nov	dec																
Number of Salmonella spp. positive cultureso	Number of samples positive for Salmonella species including typhoidal Salmonella																													
Population Strata Comments	Specify details/comments of report numerator and denominator. For traveller studies, enter the destination travelled to in this cell																													
Age information estimate	Age data reported (numeric value)		5 years; 6 months;																											
Age information estimate type	groups of age in any way (i.e. less than 15 or greater than 15); If Mean and Range are reported, mean and the standard deviation is preferred to be extracted		mean, median, range...	mean	median	range	age group	other																						
Denominator Type	Specify the denominator type as either blood culture, urine, bone marrow, Widal, or stool culture. If the outcome is diagnosed using more than one	y		Blood Culture	Urine	Stool Culture	Bone Marrow	Widal Test	Combined	Other - see comments	Test Method Unspecified	Combined with blood																		
Incidence Reported	Number of new cases of typhoid. Categorized into age adjusted, gender adjusted and overall																													
Morbidity Rate	Annual typhoid ileal perforation rate of the population. Categorized into age adjusted, gender adjusted and overall																													
Mortality Rate	Annual typhoid death rate (# deaths / # typhoid cases). Categorized into age adjusted, gender adjusted and overall																													
Performation Mortality Rate	Annual deaths from typhoid ileal perforation rate of the population. Categorized into age adjusted, gender adjusted and overall																													
Comments	Report if values were read off graph; Open text field for comments on reported variables. A place to make notes and flag issues																													
Number of AMR Samples Tested	Number of samples tested for AMR																													
MDR estimate	Multi Drug Resistant estimate, if reported in paper																													
Antimicrobial resistance rate	Prevalence rate of antimicrobial resistance reported in the study; Ensure susceptibility is converted to resistance (% resistance = 100% - % susceptibility)																													
Incidence Estimate Type				counts	rate per 1,000	rate per 10,000	rate per 100,000	rate per 1,000,000	rate per 1,000 person years	rate per 100,000 person years	rate per 1,000,000 person years	rate per 100,000 per year	rate per 100	rate per 100,000	rate per 1,000,000															
Population Surveillance Types	Passive Surveillance<br>refers to the receipt of reports of infections/disease from physicians, laboratories and other healthcare professionals. In these cases the sick people need to seek aid on their own (i.e. visit a doctor/hospital), that is, they are self-selected typhoid cases<br>Active Surveillance (this will be rare)<br>refers to a system that actively seeks people who are ill. These people would likely not have been captured otherwise. In active sampling, we do not wait for cases to be reported for for ill persons to seek care. We find them in the community and in their homes.<br>e.g. Sampling all fever cases and testing for typhoid<br>e.g. Population is defined here as say 1000 households and population sampling would be representative of these households for typhoid incidence. In this scenario study teams visited each house. Augmented Passive<br>Passive surveillance system with encouragement of screening for fevers Subjects are STILL self-selected but their participation in the surveillance system or study have been promoted with education campaigns or mass media campaigns.<br>Assisted or augmented passive sentinel surveillance helps to ensure regular reporting and to improve the data quality. Cooperation of the reporting units can be increased through a transparent reporting system, regular contact, workshops, trainings and feedback mechanisms<br>*More examples can be found at:<br>https://docs.google.com/document/d/1flNftv5iuMIctCePnTNgoVJpASk64G<br>MCb2AFtIf58cI/edit?usp=sharing			Passive Surveillance	Active Surveillance	Augmented Passive Surveillance																								
Population Types				General Population	Laboratory Studies	Ambulatory	Hospitalized																							
Overall Row			y	Stratum	Overall Monthly	AMR Only	I	D	P	Pm	ID	IP	IPm	IDP	IPPm	IDPPm	DP	DPm	DPPm	PPm										
World Bank Regions			y	Sub-Saharan Africa	Latin America and Caribbean	East Asia and Pacific	Europe and Central Asia	South Asia	Middle East and North Africa	North America	Multiple																			
Age Code			y	I	C	Y	A	S	IC	IY	IA	IS	ICY	ICA	ICS	ICYA	ICYS	ICYAS	CY	CA	CS	CYA	CYS	CYAS	YA	YS	YAS	AS	NR	?

Appendix 2.2 Reason for exclusion at full-text screening phase

**Exclusion Reason**	**Number of Studies Excluded**
No relevant outcome data/rates	425
Non-English Study	310
Data less than 18 months	231
Duplicate	129
Review	90
Developing country prior to 1990	82
Other (Math model/Graph difficult to read/vaccination study without control/human challenge)	54
Study duration Not Stated	47
Full text not found-exhausted all methods	25
Not Peer-Reviewed	20
Special populations i.e. Travellers, Military groups, Cancer, HIV positive groups, Sickle Cell Anemia groups, Outbreaks	16
Purely antimicrobial resistance reporting	13
Clinical case study/series	11
Carrier Study	10
Excluded during synthesis	296
Total Excluded	1759
