# Supplementary material for: Global Trends in Typhoidal Salmonellosis: A Systematic Review
Source: Am J Trop Med Hyg. 2018 Jul 25;99(3 Suppl):10–9. doi: 10.4269/ajtmh.18-0034 (PMC6128363; doi:10.4269/ajtmh.18-0034)
Supplement: Supplementary file 3 [file tpmd180034.SD3.pdf]

Appendix 3: List of Included Studies

Appendix 3.1: Included Peer Reviewed Studies (Blood culture confirmed enteric fever)

Author	Year of Publication	Title	Journal
Abdullah, F. E., et al.	2012	Enteric fever in karachi: Current antibiotic susceptibility of salmonellae isolates	Journal of the College of Physicians and Surgeons Pakistan 22(3): 147-150.
Abro, A. H., et al.	2012	Demographic and surgical evaluation of typhoid ileal perforation	Journal of Ayub Medical College, Abbottabad : JAMC 24(3-4): 87-89.
Abucejo, P. E., et al.	2001	Blood culture confirmed typhoid fever in a provincial hospital in the Philippines	The Southeast Asian journal of tropical medicine and public health 32(3): 531-536.
Aggarwal, A., et al.	2007	A three-year retrospective study on the prevalence, drug susceptibility pattern, and phage types of Salmonella enterica subspecies typhi and paratyphi in Christian Medical College and Hospital, Ludhiana, Punjab	Journal, Indian Academy of Clinical Medicine 8(1): 32-35.
Agu,K., et al.	2014	Prevalence, Morbidity, and Mortality Patterns of Typhoid Ileal Perforation as Seen at the University of Nigeria Teaching Hospital Enugu Nigeria: An 8-year Review	World Journal of Surgery 38(10): 2514-2518.
Al-Emran, H. M., et al.	2016	Validation and Identification of Invasive Salmonella Serotypes in Sub-Saharan Africa by Multiplex Polymerase Chain Reaction.	Clinical infectious diseases : an official publication of the Infectious Diseases Society of America 62 Suppl 1: S80-82.
Al-Emran, H. M., et al.	2016	A Multicountry Molecular Analysis of Salmonella enterica Serovar Typhi With Reduced Susceptibility to Ciprofloxacin in Sub-Saharan Africa.	Clinical infectious diseases : an official publication of the Infectious Diseases Society of America 62 Suppl 1: S42-46.
Ali, J. and Kebede, Y.	2008	Frequency of isolation and antimicrobial susceptibility pattern of bacterial isolates from blood culture, Gondar University teaching hospital, Northwest Ethiopia	Ethiopian medical journal 46(2): 155-161.
Ali, M., et al.	2011	Impact of Vi vaccination on spatial patterns of typhoid fever in the slums of Kolkata, India	Vaccine 29(48): 9051-9056.
Al-Mofleh, I.A., et al.	1990	Fever of unknown origin: Experience in Riyadh, Saudi Arabia	Annals of Saudi Medicine 10(6): 620-625.
Anonymous	1920	Typhoid in the Large Cities of the United States in 1919	JAMA 72(10): 672-675.
Aseffa, A., et al.	1997	Antibiotic resistance of prevalent salmonella and shigella strains in northwest Ethiopia	East African Medical Journal 74(11): 708-713.
Bajracharya,D., et al.	2014	25 Years after Vi Typhoid Vaccine Efficacy Study, Typhoid Affects Significant Number of Population in Nepal	PLoS ONE [Electronic Resource] 9(1).
Bhattacharya, S.S., et al.	2003	A steady decrease in occurrence of Salmonella typhi infection in Rourkela, Orissa	Indian Journal of Pathology and Microbiology 46(3): 498-500.
Bhutta,Z.A.	1996	Therapeutic aspects of typhoidal salmonellosis in childhood: The Karachi experience	Annals of Tropical Paediatrics 16(4): 299-306.
Biggs, H.M., et al.	2013	Invasive Salmonella infections in areas of high and low Malaria transmission intensity in Tanzania	Clinical Infectious Diseases 58(5): 638-647.
Blacksell, S.D., et al.	2007	Serological and blood culture investigations of Nepalese fever patients	Transactions of the Royal Society of Tropical Medicine and Hygiene 101(7): 686-690.
Breiman,R.F., et al.	2012	Population-based incidence of typhoid fever in an urban informal settlement and a rural area in Kenya: implications for typhoid vaccine use in Africa	PLoS ONE [Electronic Resource] 7(1).
Butt, T.,et al.	2005	Changing trends in drug resistance among typhoid salmonellae in Rawalpindi, Pakistan	Eastern Mediterranean Health Journal 11(5-6): 1038-1044.
Chansamouth, V., et al.	2016	The Aetiologies and Impact of Fever in Pregnant Inpatients in Vientiane, Laos	PLoS Negl Trop Dis. 2016 Apr 6;10(4):e0004577. doi: 10.1371/journal.pntd.0004577. eCollection 2016 Apr.
Cheng,A.F.B.,et al.	1991	A five-year prospective study of septicaemia in hospitalized children in Hong Kong	Journal of Tropical Medicine and Hygiene 94(5): 295-303.
Chiedozie Kingsley, O., et al.	2013	Bacteriologic profile and antibiotics susceptibility pattern of suspected septicaemic patients in Uyo, Nigeria	Research Journal of Medical Sciences 7(2): 35-39.
Ciftci, E.,et al.	2004	Salmonella bacteraemia in Turkish children: 37 cases seen in a university hospital between 1993 and 2002	Annals of Tropical Paediatrics 24(1): 75-80.
Condran, G.A. and Crimmins-Gardner, E.	1978	Public health measures and mortality in US cities in the late nineteenth century	Human Ecology 6(1): 27-54.
Cruz Espinoza, L. M., et al.	2016	Variations of Invasive Salmonella Infections by Population Size in Asante Akim North Municipal, Ghana.	Clinical infectious diseases : an official publication of the Infectious Diseases Society of America 62 Suppl 1: S17-22.
Daoud, A.S., et al.	1991	Clinical presentation of enteric fever: its changing pattern in Kuwait	Journal of Tropical Medicine and Hygiene 94(5): 341-347.
Dave, J., et al.	2015	East London experience with enteric fever 2007-2012	PloS one 10(3).
Dimitrov, T.S., et al.	2005	Incidence of bloodstream infections in a speciality hospital in Kuwait: 8-year experience	Medical Principles and Practice 14(6): 417-421.
Edino,S.T., et al.	2004	Typhoid enteric perforation in north western Nigeria	Nigerian journal of medicine : journal of the National Association of Resident Doctors of Nigeria 13(4): 345-349.
Eibach, D., et al.	2016	The Emergence of Reduced Ciprofloxacin Susceptibility in Salmonella enterica Causing Bloodstream Infections in Rural Ghana.	Clinical infectious diseases : an official publication of the Infectious Diseases Society of America 62 Suppl 1: S32-36.
Eni, U.E. and Gali, B.M.	2007	Aetiology, management and outcome of entero-cutaneous fistula in Maiduguri, Nigeria	Nigerian journal of clinical practice 10(1): 47-51.

Author	Year of Publication	Title	Journal
Fashae, K., et al.	2010	Antimicrobial susceptibility and serovars of Salmonella from chickens and humans in Ibadan, Nigeria	Journal of infection in developing countries 4(8): 484-494.
Feasey, N.A., et al.	2015	Three epidemics of invasive multidrug-resistant salmonella bloodstream infection in Blantyre, Malawi, 1998-2014	Clinical Infectious Diseases 61(pp S363-S371).
Feasey, N.A., et al.	2010	Typhoid fever and invasive nontyphoid salmonellosis, Malawi and South Africa	Emerging Infectious Diseases 16(9): 1448-1451.
Feasey, N.A., et al.	2015	Rapid Emergence of Multidrug Resistant, H58-Lineage Salmonella Typhi in Blantyre, Malawi	PLoS Neglected Tropical Diseases 9(4).
Fuerst, H.T.	1964	The epidemiology of salmonella infections in the city of New York	Bulletin of the New York Academy of Medicine 40(12): 948-960.
Gandra, S.,et al.	2015	Trends in antibiotic resistance among bacteria isolated from blood cultures using a large private laboratory network data in India: 2008- 2014	Antimicrobial Resistance and Infection Control Conference: 3rd International Conference on Prevention and Infection Control, ICPIC 2015 Geneva Switzerland. Conference Start: 20150616 Conference End: 20150619. Conference Publication: (var.pagings). 20150614 (no pagination).
Ganesh, R., et al.	2010	Profile of Typhoid Fever in Children from a Tertiary Care Hospital in Chennai-South India	Indian J Pediatr 77(10): 1089-1092.
Gautam, V., et al.	2002	Sensitivity patterns of Salmonella serotypes in Northern India	The Brazilian journal of infectious diseases : an official publication of the Brazilian Society of Infectious Diseases 6(6): 281-287.
Geetha, V.K., et al.	2014	Plasmid-mediated quinolone resistance in typhoidal Salmonellae: a preliminary report from South India	Indian J Med Microbiol 32(1): 31-34.
Guha, S., et al.	2005	Salmonella bacteraemia in Pokhara: emergence of antibiotic resistance	Nepal Medical College journal : NMCJ 7(1): 23-25.
Hafiz, S., et al.	1993	Epidemiology of salmonellosis and its sensitivity in Karachi	JPMA Journal of the Pakistan Medical Association 43(9): 178-179.
Herva,E., et al.	1999	Establishing a laboratory for surveillance of invasive bacterial infections in a tertiary care government hospital in a rural province in The Philippines	American Journal of Tropical Medicine and Hygiene 60(6): 1035-1040.
Jain, S. and Das Chugh, T.	2013	Antimicrobial resistance among blood culture isolates of Salmonella enterica in New Delhi.	Journal of infection in developing countries 7(11): 788-795.
Kalonji, L.M., et al.	2015	Invasive salmonella infections at multiple surveillance sites in the Democratic Republic of the Congo, 2011-2014	Clinical Infectious Diseases 61(pp S346-S353).
Kanoksil, M.,et al.	2013	Epidemiology, Microbiology and Mortality Associated with Community-Acquired Bacteremia in Northeast Thailand: A Multicenter Surveillance Study	PloS one 8(1).
Karkey, A.,et al.	2010	The burden and characteristics of enteric fever at a healthcare facility in a densely populated area of kathmandu	PloS one 5(11).
Kasper, M.R., et al.	2012	Infectious etiologies of acute febrile illness among patients seeking health care in South- Central Cambodia	American Journal of Tropical Medicine and Hygiene 86(2): 246-253.
Kelly, D.F., et al.	2011	The burden of vaccine-preventable invasive bacterial infections and pneumonia in children admitted to hospital in urban Nepal	International Journal of Infectious Diseases 15(1): e17-e23.
Khan, M.I., et al.	2012	Risk factors associated with typhoid fever in children aged 2-16 years in Karachi, Pakistan	Epidemiology and Infection 140(4): 665-672.
Khan, M.I., et al.	2012	Effectiveness of Vi capsular polysaccharide typhoid vaccine among children: a cluster randomized trial in Karachi, Pakistan	Vaccine 30(36): 5389-5395.
Khan, M.I., et al.	2012	Epidemiology, clinical presentation, and patterns of drug resistance of Salmonella Typhi in Karachi, Pakistan	Journal of infection in developing countries 6(10): 704-714.
Kharbanda, P., et al.	2013	Changing antimicrobial susceptibility of Salmonella isolates: A retrospective study of 3 year (2008-2010) from a tertiary care hospital	Journal of Pure and Applied Microbiology 7(2): 1455-1456.
Kouame, J., et al.	2004	Typhoid ileal perforation surgical experience of 64 cases	Acta Chirurgica Belgica 104(4): 445-447.
Kuchuloria, T., et al.	2016	Hospital-Based Surveillance for Infectious Etiologies among Patients with Acute Febrile Illness in Georgia, 2008-2011	Am. J. Trop. Med. Hyg., 94(1), 2016, pp. 236–242
Labi, A.K., et al.	2014	Salmonella blood stream infections in a tertiary care setting in Ghana	BMC infectious diseases 14.
Laupland, K.B., et al.	2010	Salmonella enterica bacteraemia: a multi-national population-based cohort study	BMC infectious diseases 10.
Leelarasamee, A., et al.	2004	Etiologies of acute undifferentiated febrile illness in Thailand	Journal of the Medical Association of Thailand 87(5): 464-472.
Limpitikul, W., et al.	2014	Typhoid outbreak in Songkhla, Thailand 2009-2011: clinical outcomes, susceptibility patterns, and reliability of serology tests.	PloS one 9(11): e111768.
Lin, F.Y.C., et al.	2001	The efficacy of a Salmonella typhi Vi conjugate vaccine in two-to-five-year-old children	New England Journal of Medicine 344(17): 1263-1269.
Lunguya, O., et al.	2012	Salmonella typhi in the democratic republic of the congo: fluoroquinolone decreased susceptibility on the rise	PLoS Neglected Tropical Diseases 6(11).
Lunguya, O., et al.	2012	The diagnosis of typhoid fever in the Democratic Republic of the Congo	Transactions of the Royal Society of Tropical Medicine and Hygiene 106(6): 348-355.
Mandomando, I., et al.	2015	Invasive salmonella infections among children from Rural Mozambique, 2001-2014	Clinical Infectious Diseases 61(pp S339-S345).
Maskey, A.P., et al.	2008	Emerging trends in enteric fever in Nepal: 9124 cases confirmed by blood culture 1993-2003	Transactions of the Royal Society of Tropical Medicine and Hygiene 102(1): 91- 95.
Mayanja, B.N., et al.	2010	Septicaemia in a population-based HIV clinical cohort in rural Uganda, 1996-2007: incidence, aetiology, antimicrobial drug resistance and impact of antiretroviral therapy	Tropical Medicine & International Health 15(6): 697-705.
Meier, D.E. and Tarpley, J.L.	1998	Typhoid intestinal perforations in Nigerian children	World Journal of Surgery 22(3): 319-323.
Meltzer, E., et al.	2006	Epidemiology and clinical aspects of enteric fever in Israel	American Journal of Tropical Medicine and Hygiene 74(4): 540-545.
Menezes, G.A., et al.	2012	Antimicrobial resistance trends in blood culture positive Salmonella Typhi isolates from Pondicherry, India, 2005-2009	Clinical Microbiology and Infection 18(3): 239-245.
Mouaket, A.E., et al.	1990	Prolonged unexplained pyrexia: A review of 221 paediatric cases from Kuwait	Infection 18(4): 226-229.

Author	Year of Publication	Title	Journal
Mutanda,L.N.,et al.	1998	Selected laboratory tests in febrile patients in Kampala, Uganda	East African Medical Journal 75(2): 68-72.
Muthumbi,E., et al.	2015	Invasive Salmonellosis in Kilifi, Kenya	Clinical Infectious Diseases 61 Suppl 4: S290-301.
Neilsen, M.V., et al.	2012	Incidence and characteristics of bacteremia among children in rural Ghana	PloS one 7(9).
Ng, S.Y. and Leong,K.W.	1955	Typhoid fever in Singapore	Proc Alumni. Ass. Malaya 8(3): 183-189.
Obaro,S.K., et al.	2015	Salmonella bacteremia among children in central and Northwest Nigeria, 2008-2015	Clinical Infectious Diseases 61(pp S325-S331).
Ochiai, R.L., et al.	2008	A study of typhoid fever in five Asian countries: Disease burden and implications for controls	Bulletin of the World Health Organization 86(4): 260-268.
Phoba, M.F., et al.	2014	Epidemic increase in Salmonella bloodstream infection in children, Bwamanda, the Democratic Republic of Congo	European Journal of Clinical Microbiology & Infectious Diseases 33(1): 79-87.
Prasad, K.J., et al.	2015	Comparative evaluation of two rapid Salmonella-IgM tests and blood culture in the diagnosis of enteric fever	Indian Journal of Medical Microbiology 33(2): 237-242.
Ravikumar,K., et al.	2006	A two-year epidemiological study on enteric fever caused by salmonella sp from Tamil Nadu, South India	Asian Journal of Microbiology, Biotechnology and Environmental Sciences 8(4): 795-797.
Raza, A., et al.	2014	Salmonella bacteraemia among healthcare workers and their dependents	Journal of the Pakistan Medical Association 64(7): 748-750.
Saha, M.R., et al.	2002	Decreasing trend in the occurence of salmonella enterica serotype typhi amongst hospitalised children in Kolkata, India during 1990-2000	Indian Journal of Medical Research 115(FEB.): 46-48.
Saha, M.R., et al.	2003	A note on incidence of typhoid fever in diverse age groups in Kolkata, India	Japanese Journal of Infectious Diseases 56(3): 121-122.
Saha, S.K., et al.	2001	Typhoid fever in Bangladesh: implications for vaccination policy	Pediatric Infectious Disease Journal 20(5): 521-524.
Schwarz, N.G., et al.	2010	Systemic bacteraemia in children presenting with clinical pneumonia and the impact of non- typhoid salmonella (NTS)	BMC infectious diseases 10.
Sharma, N.P., et al.	2006	A hospital-based study of bloodstream infections in febrile patients in Dhulikhel Hospital Kathmandu University Teaching Hospital, Nepal	Southeast Asian Journal of Tropical Medicine and Public Health 37(2): 351-356.
Siddiqui, F.J., et al.	2006	Typhoid fever in children: some epidemiological considerations from Karachi, Pakistan	International Journal of Infectious Diseases 10(3): 215-222.
Singh, D.S., et al.	2012	Enteric fever in children at Dhulikhel hospital	Journal of Nepal Paediatric Society 32(3): 216-220.
Sridhar, C.B. and Kulkarni, R.D.	1995	Reassessment of frequency of occurrence of typhoid fever and cost efficacy analysis of antibiotic therapy	The Journal of the Association of Physicians of India 43(10): 679-684.
Stephen, J.M., et al.	2003	Salmonella bloodstream infections: report from the SENTRY Antimicrobial Surveillance Program (1997-2001)	International Journal of Antimicrobial Agents 22(4): 395-405.
Stoesser, N., et al.	2013	Pediatric bloodstream infections in Cambodia, 2007 to 2011	Pediatric Infectious Disease Journal 32(7): e272-e276.
Sur, D., et al.	2009	A cluster-randomized effectiveness trial of vi typhoid vaccine in India	New England Journal of Medicine 361(4): 335-344.
Talpur, A.A., et al.	2012	Sensitivity pattern of salmonella typhi in patients with typhoid small bowel perforations	Medical Channel 19(2): 93-96.
Tankhiwale, S. S., et al.	2003	An unusually high occurrence of Salmonella enterica serotype Paratyphi A in patients with enteric fever	Indian Journal of Medical Research 117: 10-12.
Thriemer,K., et al.	2012	The Burden of Invasive Bacterial Infections in Pemba, Zanzibar	PloS one 7(2).
Vlieghe,E.R., et al.	2013	Bloodstream Infection among Adults in Phnom Penh, Cambodia: Key Pathogens and Resistance Patterns	PloS one 8(3).
Walia, M., et al.	2006	Age-related clinical and microbiological characteristics of enteric fever in India	Transactions of the Royal Society of Tropical Medicine and Hygiene 100(10): 942-948.
Walters, M.S., et al.	2014	Shifts in Geographic Distribution and Antimicrobial Resistance during a Prolonged Typhoid Fever Outbreak - Bundibugyo and Kasese Districts, Uganda, 2009-2011	PLoS Neglected Tropical Diseases 8(3).
Wongsawat, J., et al.	2002	Typhoid fever in children: Experience in King Chulalongkorn Memorial Hospital	Journal of the Medical Association of Thailand 85(12): 1247-1250.
Yashavanth, R. and Vidyalakshmi, K.	2010	The Re-Emergence of chloramphenicol sensitivity among enteric fever pathogens in Mangalore	Journal of Clinical and Diagnostic Research 4(5): 3016-3108.

Appendix 3.2: Included Surveillance Reports

Author	Year of Publication	Title	Journal	Country	Level of reporting
Keddy	2007	GERMS- South Africa Annual Report 2006	National Institute for Communicable Diseases	South Africa	country-wide
Cohen	2008	GERMS- South Africa Annual Report 2007	National Institute for Communicable Diseases	South Africa	country-wide
Cohen	2009	GERMS- South Africa Annual Report 2008	National Institute for Communicable Diseases	South Africa	country-wide
Crowther	2010	GERMS- South Africa Annual Report 2009	National Institute for Communicable Diseases	South Africa	country-wide
Crowther	2011	GERMS- South Africa Annual Report 2010	National Institute for Communicable Diseases	South Africa	country-wide
Crowther-Gibson	2012	GERMS- South Africa Annual Report 2011	National Institute for Communicable Diseases	South Africa	country-wide
Eramus	2013	GERMS- South Africa Annual Report 2012	National Institute for Communicable Diseases	South Africa	country-wide
Crowther-Gibson	2014	GERMS- South Africa Annual Report 2013	National Institute for Communicable Diseases	South Africa	country-wide
Keddy	2006	Communicable Diseases Surveillance Bulletin January 2005	National Institute for Communicable Diseases	South Africa	country-wide
Keddy	2007	Communicable Diseases Surveillance Bulletin March 2006	National Institute for Communicable Diseases	South Africa	country-wide
Keddy	2015	Communicable Diseases Surveillance Bulletin April 2015	National Institute for Communicable Diseases	South Africa	country-wide
Rios	2015	Boletín Epidemiológico Trimestral Enero a Marzo de 2015- Chile	Boletín Epidemiológico Trimestral	Chile	country-wide
Palihawadana	2015	Typhoid Vaccination Situation in Sri Lanka	Ministry of Health, Sri Lanka	Sri Lanka	country-wide
MOH-NEPAL	2009	Annual Report- Department of Health Services 2009-2010	Government of Nepal, Ministry of Health and Population, Department of Health Services, Kathmandu	Nepal	country-wide
MOH-NEPAL	2010	Annual Report- Department of Health Services 2010-2011	Government of Nepal, Ministry of Health and Population, Department of Health Services, Kathmandu	Nepal	country-wide
MOH-NEPAL	2011	Annual Report- Department of Health Services 2011-2012	Government of Nepal, Ministry of Health and Population, Department of Health Services, Kathmandu	Nepal	country-wide
MOH-NEPAL	2012	Annual Report- Department of Health Services 2012-2013	Government of Nepal, Ministry of Health and Population, Department of Health Services, Kathmandu	Nepal	country-wide
MOH-NEPAL	2013	Annual Report- Department of Health Services 2013-2014	Government of Nepal, Ministry of Health and Population, Department of Health Services, Kathmandu	Nepal	country-wide
WHO-IRAQ	2008	Integrated Control of Communicable Diseases	WHO	Iraq	country-wide
MOH-BAHRAIN	2001	Health Statistics Report 2000	Health Information Directorate	Bahrain	country-wide
MOH-BAHRAIN	2002	Health Statistics Report 2001	Health Information Directorate	Bahrain	country-wide
MOH-BAHRAIN	2003	Health Statistics Report 2002	Health Information Directorate	Bahrain	country-wide
MOH-BAHRAIN	2004	Health Statistics Report 2003	Health Information Directorate	Bahrain	country-wide
MOH-BAHRAIN	2005	Health Statistics Report 2004	Health Information Directorate	Bahrain	country-wide
MOH-BAHRAIN	2006	Health Statistics Report 2005	Health Information Directorate	Bahrain	country-wide
MOH-BAHRAIN	2007	Health Statistics Report 2006	Health Information Directorate	Bahrain	country-wide
MOH-BAHRAIN	2008	Health Statistics Report 2007	Health Information Directorate	Bahrain	country-wide
MOH-BAHRAIN	2009	Health Statistics Report 2008	Health Information Directorate	Bahrain	country-wide
MOH-BAHRAIN	2010	Health Statistics Report 2009	Health Information Directorate	Bahrain	country-wide
MOH-BAHRAIN	2011	Health Statistics Report 2010	Health Information Directorate	Bahrain	country-wide
MOH-BAHRAIN	2012	Health Statistics Report 2011	Health Information Directorate	Bahrain	country-wide
MOH-BAHRAIN	2013	Health Statistics Report 2012	Health Information Directorate	Bahrain	country-wide
MOH-BAHRAIN	2014	Health Statistics Report 2013	Health Information Directorate	Bahrain	country-wide
MOH-GHANA	2007	GHS Facts and Figures 2007	MOH-GHANA	Ghana	country-wide
MOH-GHANA	2010	GHS Facts and Figures 2010	MOH-GHANA	Ghana	country-wide
MOH-QATAR	1981	Annual Report 1981	MOH-QATAR	Qatar	country-wide
MOH-QATAR	1982	Annual Report 1982	MOH-QATAR	Qatar	country-wide
MOH-QATAR	1983	Annual Report 1983	MOH-QATAR	Qatar	country-wide
MOH-QATAR	1984	Annual Report 1984	MOH-QATAR	Qatar	country-wide
MOH-QATAR	1985	Annual Report 1985	MOH-QATAR	Qatar	country-wide
MOH-QATAR	1986	Annual Report 1986	MOH-QATAR	Qatar	country-wide
MOH-QATAR	1987	Annual Report 1987	MOH-QATAR	Qatar	country-wide
MOH-QATAR	1988	Annual Report 1988	MOH-QATAR	Qatar	country-wide
MOH-QATAR	1989	Annual Report 1989	MOH-QATAR	Qatar	country-wide
MOH-QATAR	1990	Annual Report 1990	MOH-QATAR	Qatar	country-wide
MOH-QATAR	1991	Annual Report 1991	MOH-QATAR	Qatar	country-wide
MOH-QATAR	1992	Annual Report 1992	MOH-QATAR	Qatar	country-wide
MOH-QATAR	1993	Annual Report 1993	MOH-QATAR	Qatar	country-wide
MOH-QATAR	1994	Annual Report 1994	MOH-QATAR	Qatar	country-wide
MOH-QATAR	1995	Annual Report 1995	MOH-QATAR	Qatar	country-wide
MOH-QATAR	1996	Annual Report 1996	MOH-QATAR	Qatar	country-wide
MOH-QATAR	1997	Annual Report 1997	MOH-QATAR	Qatar	country-wide
MOH-QATAR	1998	Annual Report 1998	MOH-QATAR	Qatar	country-wide
MOH-QATAR	1999	Annual Report 1999	MOH-QATAR	Qatar	country-wide
MOH-QATAR	2000	Annual Report 2000	MOH-QATAR	Qatar	country-wide
MOH-QATAR	2001	Annual Report 2001	MOH-QATAR	Qatar	country-wide
MOH-QATAR	2002	Annual Report 2002	MOH-QATAR	Qatar	country-wide
MOH-QATAR	2003	Annual Report 2003	MOH-QATAR	Qatar	country-wide
MOH-QATAR	2004	Annual Report 2004	MOH-QATAR	Qatar	country-wide
MOH-QATAR	2005	Annual Report 2005	MOH-QATAR	Qatar	country-wide
MOH-QATAR	2006	Annual Report 2006	MOH-QATAR	Qatar	country-wide
MOH-QATAR	2007	Annual Report 2007	MOH-QATAR	Qatar	country-wide
MOH-QATAR	2008	Annual Report 2008	MOH-QATAR	Qatar	country-wide

Author	Year of Publication	Title	Journal	Country	Level of reporting
MOH-QATAR	2009	Annual Report 2009	MOH-QATAR	Qatar	country-wide
MOH-QATAR	2010	Annual Report 2010	MOH-QATAR	Qatar	country-wide
MOH-QATAR	2011	Annual Report 2011	MOH-QATAR	Qatar	country-wide
MOH-QATAR	2012	Annual Report 2012	MOH-QATAR	Qatar	country-wide
Sharma	2007	Surveillance of communicable diseases in tertiary health care system in Chandigarh, UT	Indian journal of medical sciences	India	Chandigarh
Ratnawati	2008	Enteric fever in endemic areas of Indonesia: an increasing problem of resistance	Hasanuddin University, Makassar, Indonesia	Indonesia	country-wide
Thong	2005	Surveillance and Surveillance and Subtyping Subtyping of Salmonella Salmonella spp. in Malaysia.	University of Malaysia	Malaysia	country-wide
MOPH-THAILAND	2014	Annual Report- Bureau of Epidemiology- Typhoid	Ministry of Public Health	Thailand	country-wide
MOPH-THAILAND	2014	Annual Report- Bureau of Epidemiology- Paratyphoid	Ministry of Public Health	Thailand	country-wide
CDC-TAIWAN	2006	Statistcs of communicable diseases and surveillance report in Taiwan area, 2004	Centre for Disease Control Department of Health Executive Yuan	Taiwan, China	country-wide
MOH-CAMBODIA	2007	Annual Health Statistics Cambodia 2006	Department of Planning and Health Information	Cambodia	country-wide
MOH-CAMBODIA	2008	Annual Health Statistics Cambodia 2007	Department of Planning and Health Information	Cambodia	country-wide
MOH-CAMBODIA	2009	Annual Health Statistics Cambodia 2008	Department of Planning and Health Information	Cambodia	country-wide
MOH-CAMBODIA	2010	Annual Health Statistics Cambodia 2009	Department of Planning and Health Information	Cambodia	country-wide
MOH-CAMBODIA	2011	Annual Health Statistics Cambodia 2010	Department of Planning and Health Information	Cambodia	country-wide
MOH-CAMBODIA	2012	Annual Health Statistics Cambodia 2011	Department of Planning and Health Information	Cambodia	country-wide
Hargreaves	1995	Annual report of the national notifiable diseases surveillance system, 1994	Annual report of the National Notifiable Diseases Surveillance System	Australia	country-wide
Herceg	1996	Annual report of the national notifiable diseases surveillance system, 1995	Annual report of the National Notifiable Diseases Surveillance System	Australia	country-wide
Curran	1997	Australia’s notifiable diseases status, 1996	Annual report of the National Notifiable Diseases Surveillance System	Australia	country-wide
Blumer	2002	Annual report of the national notifiable diseases surveillance system, 1994	Annual report of the National Notifiable Diseases Surveillance System	Australia	country-wide
Begg	2006	Australia's notifiable diseases status, 2006	Annual report of the National Notifiable Diseases Surveillance System	Australia	country-wide
Corvisy	2013	Australia’s notifiable disease status, 2012	Annual report of the National Notifiable Diseases Surveillance System	Australia	country-wide
MOH- NEW ZEALAND	2003	Notifiable disease surveillance	New Zealand Public Health Surveillance Report	New Zealand	country-wide
MOH- NEW ZEALAND	2004	Notifiable disease surveillance	New Zealand Public Health Surveillance Report	New Zealand	country-wide
MOH- NEW ZEALAND	2005	Notifiable disease surveillance	New Zealand Public Health Surveillance Report	New Zealand	country-wide
MOH- NEW ZEALAND	2007	2. Notifiable disease surveillance	New Zealand Public Health Surveillance Report	New Zealand	country-wide
MOH- NEW ZEALAND	2008	Notifiable disease surveillance	New Zealand Public Health Surveillance Report	New Zealand	country-wide
MOH- NEW ZEALAND	2009	Notifiable disease surveillance	New Zealand Public Health Surveillance Report	New Zealand	country-wide
MOH- NEW ZEALAND	2010	Notifiable disease surveillance	New Zealand Public Health Surveillance Report	New Zealand	country-wide
MOH- NEW ZEALAND	2011	Notifiable Disease Surveillance	New Zealand Public Health Surveillance Report	New Zealand	country-wide
MOH- NEW ZEALAND	2012	Outbreak surveillance	New Zealand Public Health Surveillance Report	New Zealand	country-wide
Minesterio De Salud Publica	2013	Anuario Estadistico de Salud	Ministerio de Salud Publica Dirreccion Nacional de Registro Medicos Y Estadisticas de Salud	Cuba	country-wide
Minesterio De Salud Publica	2016	Anuario Estadistico de Salud	Ministerio de Salud Publica Dirreccion Nacional de Registro Medicos Y Estadisticas de Salud	Cuba	country-wide
Robert Koch Institut	2001	Infektionsepidemiologisches Jahrbuch meldepflichtiger Krankheiten für 2001		Germany	country-wide
Robert Koch Institut	2002	Infektionsepidemiologisches Jahrbuch meldepflichtiger Krankheiten für 2002		Germany	country-wide
Robert Koch Institut	2003	Infektionsepidemiologisches Jahrbuch meldepflichtiger Krankheiten für 2003		Germany	country-wide
Robert Koch Institut	2004	Infektionsepidemiologisches Jahrbuch meldepflichtiger Krankheiten für 2004		Germany	country-wide
Robert Koch Institut	2005	Infektionsepidemiologisches Jahrbuch meldepflichtiger Krankheiten für 2005		Germany	country-wide
Robert Koch Institut	2006	Infektionsepidemiologisches Jahrbuch meldepflichtiger Krankheiten für 2006		Germany	country-wide
Robert Koch Institut	2007	Infektionsepidemiologisches Jahrbuch meldepflichtiger Krankheiten für 2007		Germany	country-wide

Author	Year of Publication	Title	Journal	Country	Level of reporting
Robert Koch Institut	2008	Infektionsepidemiologisches Jahrbuch meldepflichtiger Krankheiten für 2008		Germany	country-wide
Robert Koch Institut	2009	Infektionsepidemiologisches Jahrbuch meldepflichtiger Krankheiten für 2009		Germany	country-wide
Robert Koch Institut	2010	Infektionsepidemiologisches Jahrbuch meldepflichtiger Krankheiten für 2010		Germany	country-wide
Robert Koch Institut	2011	Infektionsepidemiologisches Jahrbuch meldepflichtiger Krankheiten für 2011		Germany	country-wide
Robert Koch Institut	2012	Infektionsepidemiologisches Jahrbuch meldepflichtiger Krankheiten für 2012		Germany	country-wide
Robert Koch Institut	2013	Infektionsepidemiologisches Jahrbuch meldepflichtiger Krankheiten für 2013		Germany	country-wide
Robert Koch Institut	2014	Infektionsepidemiologisches Jahrbuch meldepflichtiger Krankheiten für 2014		Germany	country-wide
Department of Health EPIDEMIOLOGY BUREAU	2002	The 2001 Philippines Health Statistics		Philippines	country-wide
Department of Health EPIDEMIOLOGY BUREAU	2012	The 2011 Philippines Health Statistics		Philippines	country-wide
Lutui		Typhoid fever in tonga		Tonga	Country-wide
CDC -USA	2013	Typhoid and Paratyphoid Fever Annual Summary 2013	Nationally Notifiable Disease Surveillance System (NNDSS)	USA	Country-wide
CDC -USA	2015	Summary of Notifiable Infectious Diseases and Conditions — United States, 2013	Morbidity and Mortality Weekly Report	USA	Country-wide

Appendix 3.3: Included Conference Proceedings (Blood culture confirmed enteric fever)

Author	Year of Publication	Title	Journal
Andrews,J	2015	High rates of enteric fever diagnosis and low burden of disease in rural Nepal	Coalition Against Typhoid 9th Conference Proceedings.
Levine,M	2013	An Overview of iNTS.	Coalition Against Typhoid 8th Conference Proceedings.
Menezes, G.A., et al.	2012	Antimicrobial resistance trends in blood culture positive Salmonella Paratyphi A isolates from Pondicherry, India, 2005 - 2009	International Journal of Infectious Diseases June 2012;16():e425 2012 June
Rudiman, P.I., et al.	2009	The etiologies of acute undifferentiated febrile illness in an adult cohort in Bandung, Indonesia (2000-2008)	American Journal of Tropical Medicine and Hygiene November 2009;1)():319 2009 November
Saha,S	2013	Pediatric infection with typhoid fever in Bangladesh	Coalition Against Typhoid 8th Conference Proceedings.
Thompson, C	2015	The growing burden of S. Paratyphi A in Kathmandu- epidemiological observations from a decade of clinical trials.	Coalition Against Typhoid 9th Conference Proceedings.
