# Supplementary material for: Global Trends in Typhoidal Salmonellosis: A Systematic Review
Source: Am J Trop Med Hyg. 2018 Jul 25;99(3 Suppl):10–9. doi: 10.4269/ajtmh.18-0034 (PMC6128363; doi:10.4269/ajtmh.18-0034)

Appendix 4: Typhoid and Paratyphoid Regional Incidence Trends (per 100, 000 population) from Surveillance or the General Population

Appendix 4.1: Typhoid Regional Incidence Trends (per 100, 000 population) – Europe and Central Asia

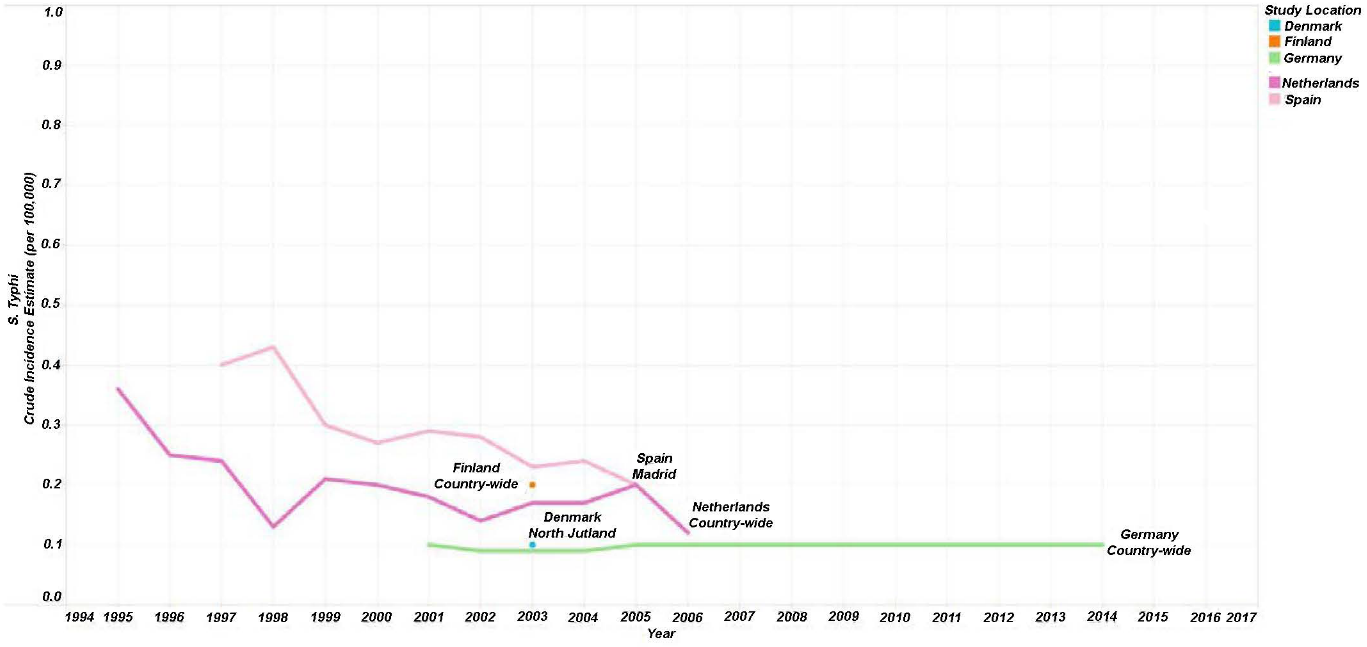

Appendix 4.2: Typhoid Regional Incidence Trends (per 100, 000 population) – Latin America & the Caribbean

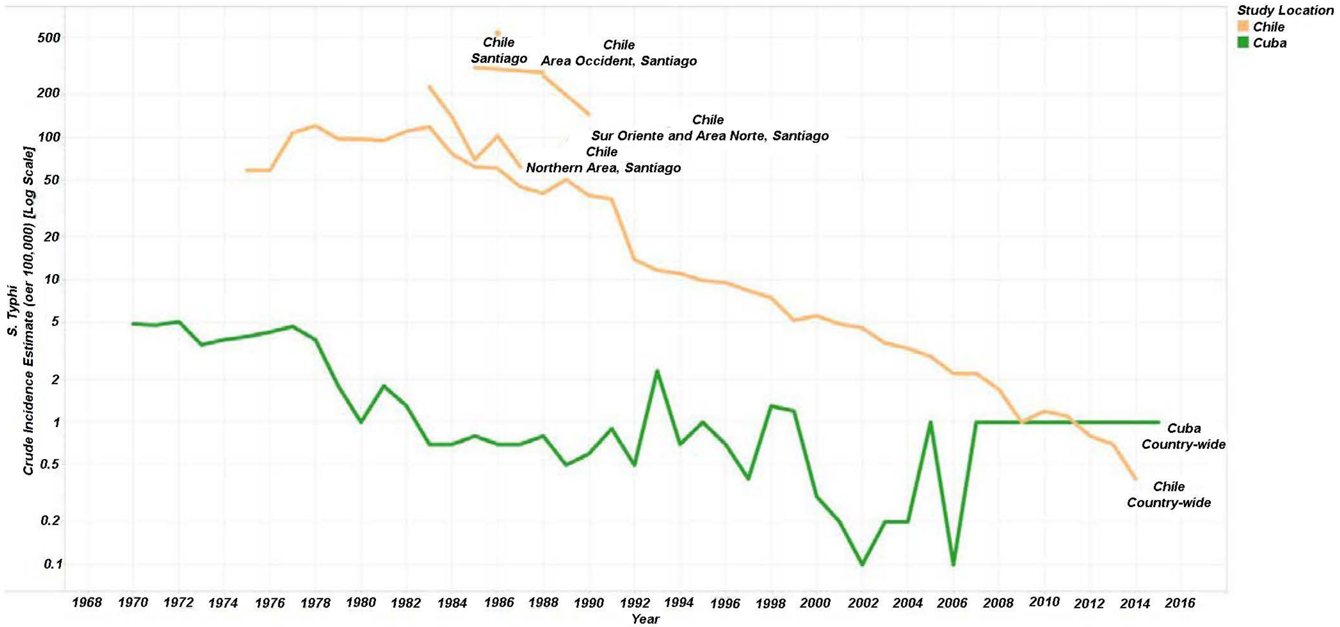

Appendix 4.3: Typhoid Regional Incidence Trends (per 100, 000 population) – North America

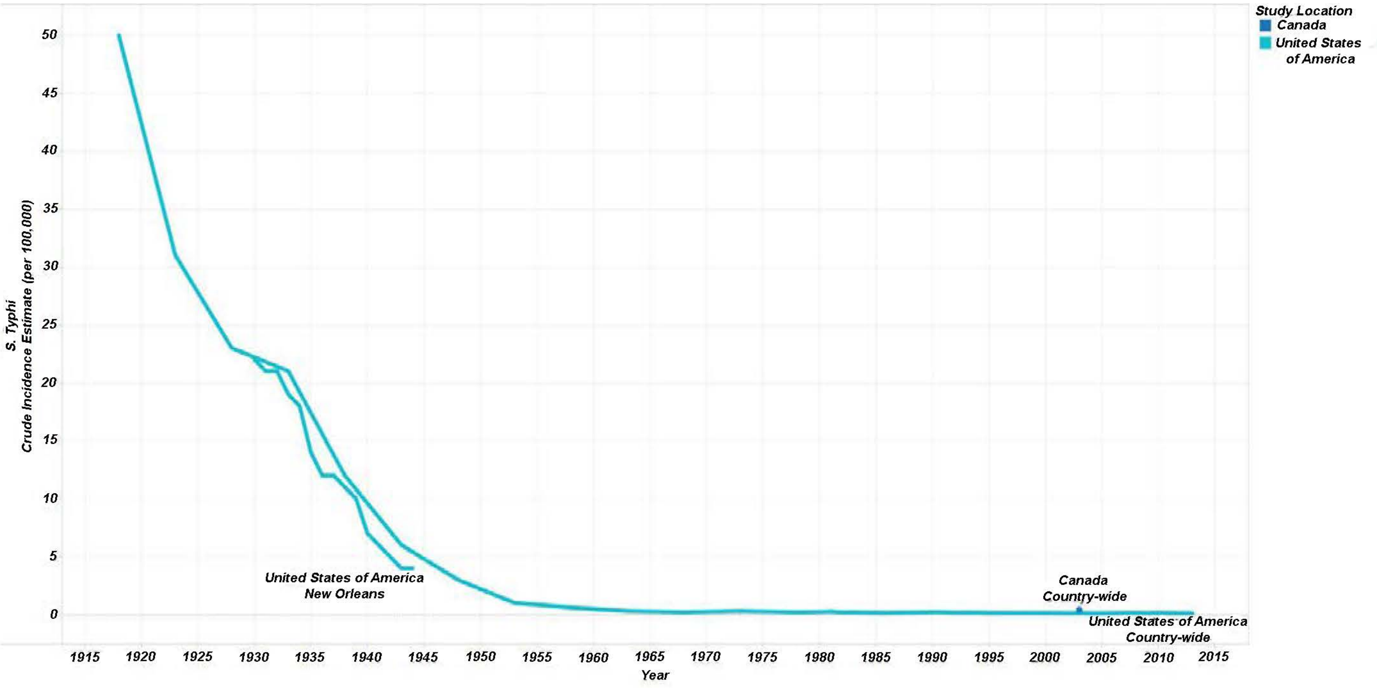

Appendix 4.4: Paratyphoid Incidence Trends (per 100, 000 population) – All Regions

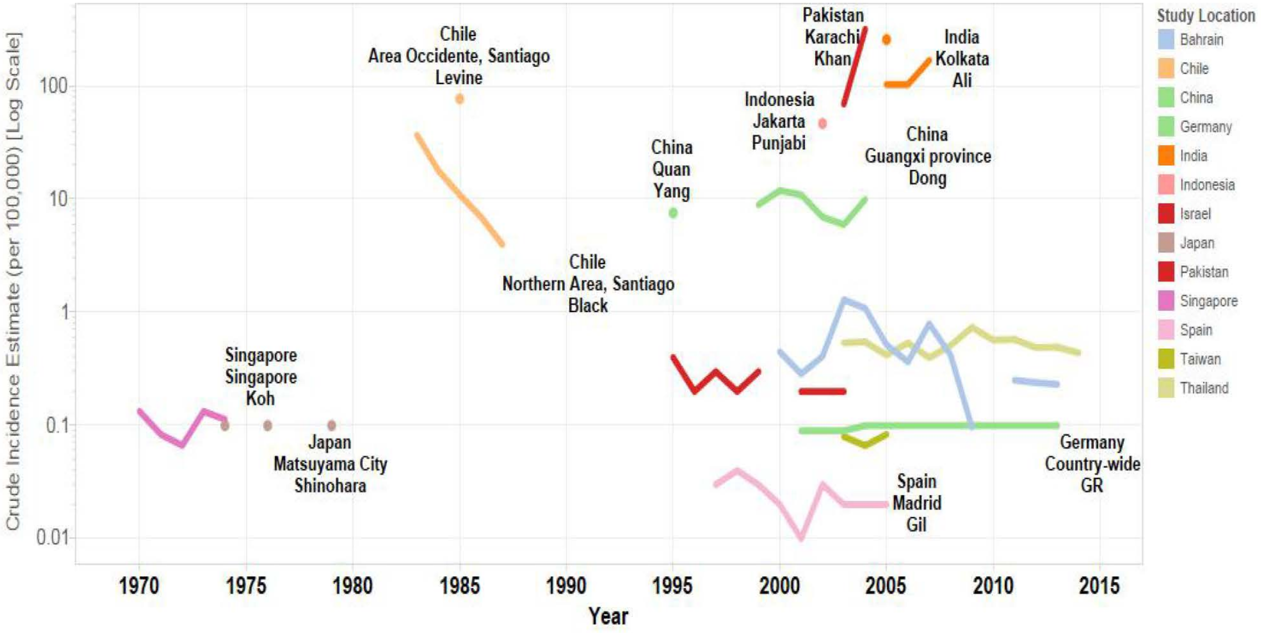

Supplement: Supplementary file 4 [file tpmd180034.SD4.pdf]
