# Supplementary material for: Global Trends in Typhoidal Salmonellosis: A Systematic Review
Source: Am J Trop Med Hyg. 2018 Jul 25;99(3 Suppl):10–9. doi: 10.4269/ajtmh.18-0034 (PMC6128363; doi:10.4269/ajtmh.18-0034)

Appendix 5: Regional Prevalence’s of Typhoid and Paratyphoid Fevers

Appendix 5.1 South Asia S. Typhi Blood Culture Prevalence

Country	Location	From	To	Author	Year	Sample
Bangladesh	Dhaka	1994	2011	Saha	2002	97065
		1998	1999	Saha	1999	4650
India	Bangalore	1990	1992	Sridhar	1990	27027
					1991	29105
					1992	22098
	Chennai	2005	2008	Ganesh	2007	45196
	Coimbatore	2003	2004	Ravikumar	2003	114
					2004	95
	Country-wide	2008	2014	Gandra	2011	136915
	Kolkata	1990	2000	Saha	1990	470
					1991	260
					1992	120
					1993	100
					1994	80
					1995	30
					1996	50
					1997	50
					1998	100
					1999	110
					2000	110
			2002	Saha	1996	1736
	Ludhiana	2003	2005	Aggarwal	2003	4500
					2004	5204
					2005	6571
	Mangalore	2005	2007	Yashavanth	2007	6297
	New Delhi	2008	2010	Kharbanda	2009	24091
			2012	Prasad	2010	2699
		2010	2012	Jain	2011	13672
	Puducherry	2005	2009	Menezes	2007	3744
		2010	2012	Geetha	2012	14050
	Rohtak	1997	2001	Gautam	1997	1167
					1998	1101
					1999	1162
					2000	1743
					2001	1783
	Rourkela	1999	2001	Bhattacharya	2000	4378
Nepal	Country-wide	2013	2015	Andrews	2014	1821
	Kathmandu	1993	2003	Maskey	1993	6065
					1994	6268
					1995	6643
					1996	6124
					1997	5308
					1998	6025
					1999	6178
					2000	5928
					2001	9059
					2002	7211
					2003	17658
		2002	2004	Blacksell	2003	103
				Sharma	2003	1774
		2005	2006	Kelly	2006	1897
			2009	Karkey	2005	14295
					2006	14018
					2007	12354
					2008	13869
			2014	Thompson	2009	2118
		2008	2012	Bajracharya	2008	4474
					2009	5762
					2010	7303
					2011	9466
					2012	3581
		2009	2011	Singh	2010	138
	Pokhara	2000	2003	Guha	2001	2354
Pakistan	Hyderabad	2007	2010	Talpur	2009	187
	Karachi	1987	1991	Hafiz	1990	6153
					1991	6292
		1991	2001	Siddiqui	2000	11668
		2008	2010	Abdullah	2009	481
	Lahore	2007	2011	Raza	2007	332
					2008	350
					2009	309
					2010	486
					2011	1055
	Rawalpindi	1996	2003	Butt	1996	3906
					1997	3737
					1998	3670
					1999	3048
					2000	3583
					2001	3866
					2002	3872
					2003	3422

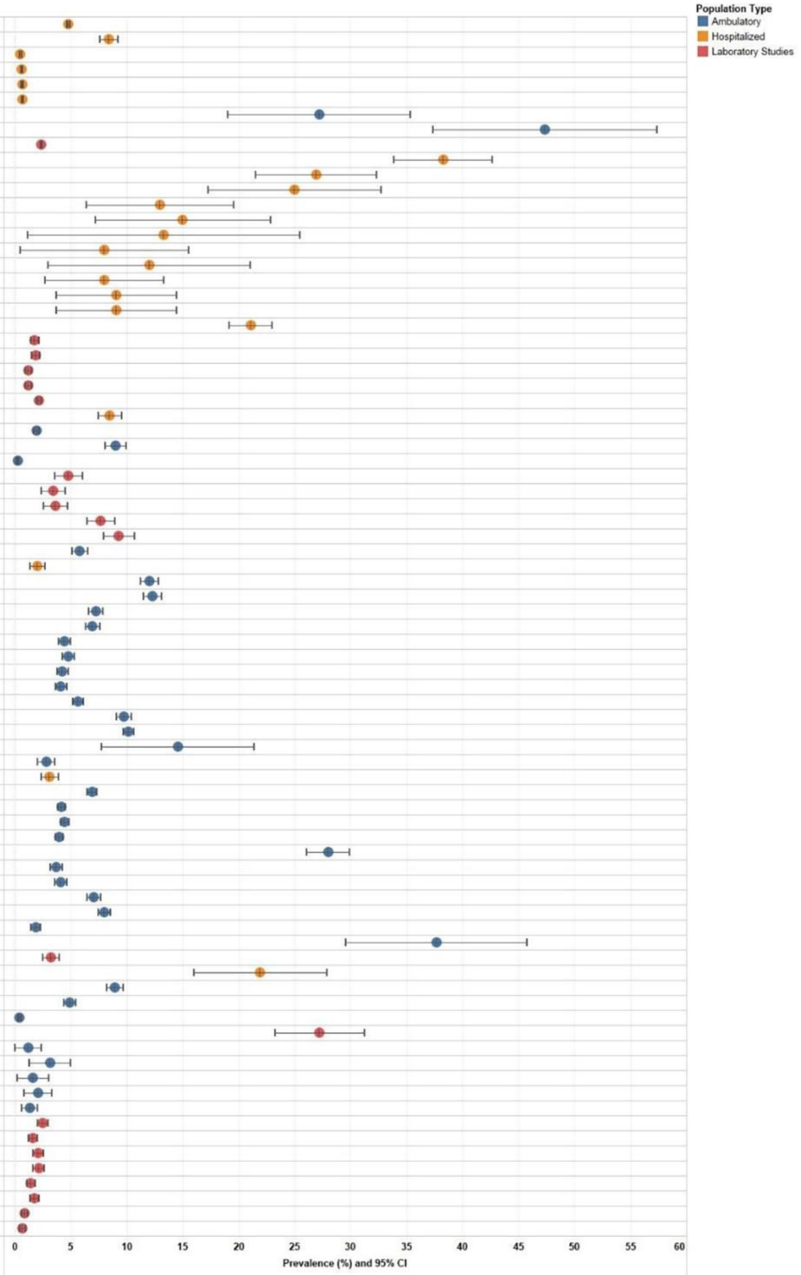

Appendix 5.2 Sub-Saharan Africa S. Typhi Blood Culture Prevalence

Country	Location	From	To	Author	Year	Sample
Burkina Faso	Nikoko and Polesgo	2011	2013	Al-Emran	2012	1674
Congo, Dem. Rep	Country-wide	2007	2009	Lunguya	2008	6677
			2011	Lunguya	2009	9634
	Kinshasa, Equateur, Orientale, and Bas-Congo	2011	2014	Kalonji	2011	2896
					2012	3176
					2013	2970
					2014	4986
	Libreville	2008	2011	Phoba	2010	331
Ethiopia	Gondar	1994	1996	Aseffa	1995	9356
		2001	2005	Ali	2003	472
Ghana	Accra	2010	2013	Labi	2012	23708
	Agogo	2007	2008	Marks	2008	1456
			2009	Neilsen	2008	1196
				Schwarz	2008	1032
			2012	Eibach	2010	5211
	Ashanti Region	2010	2012	CruzEspinoza	2011	2651
	Country-wide	2010	2013	Al-Emran	2012	3726
Guinea-Bissau	Bandim	2011	2013	Al-Emran	2012	1021
Kenya	Country-wide	2006	2009	Breiman	2008	5977
	Kibera	2011	2013	Al-Emran	2012	1251
	Kilifi	1998	2014	Muthumbi	2006	71820
Madagascar	Imerintsiatosika and Isotry	2011	2013	Al-Emran	2012	2477
Malawi	Blantyre	1998	2014	Feasey	1998	8545
					1999	7823
					2000	8052
					2001	7653
					2002	8978
					2003	11174
					2004	10653
					2005	12933
					2006	10123
					2007	9167
					2008	8628
					2009	7991
					2010	8507
					2011	9890
					2012	10433
					2013	12815
					2014	13663
	Blantyre District	1998	2014	Feasey	2011	9890
Mali	Bamako	2002	2012	Levine	2007	23151
Mozambique	Manhiça	2001	2014	Mandomando	2008	41668
Nigeria	Ibadan province	2004	2005	Fashae	2005	991
	Northwest and Central Nigeria	2008	2015	Obaro	2012	1573
	Uyo	2011	2012	Chiedozie Kingsley	2012	689
Senegal	Pikine	2011	2013	Al-Emran	2012	1058
Tanzania	Moshi	2006	2008	Biggs	2007	4106
		2011	2013	Al-Emran	2012	680
	Muheza District	2011	2012	Hildenwall	2012	1028
	Pemba	2009	2010	Thriemer	2010	2209
Uganda	Country-wide	1996	2007	Mayanja	2001	703
	Kampala	1992	1996	Mutanda	1992	1009
					1993	1005
					1994	1036
					1995	413
					1996	443

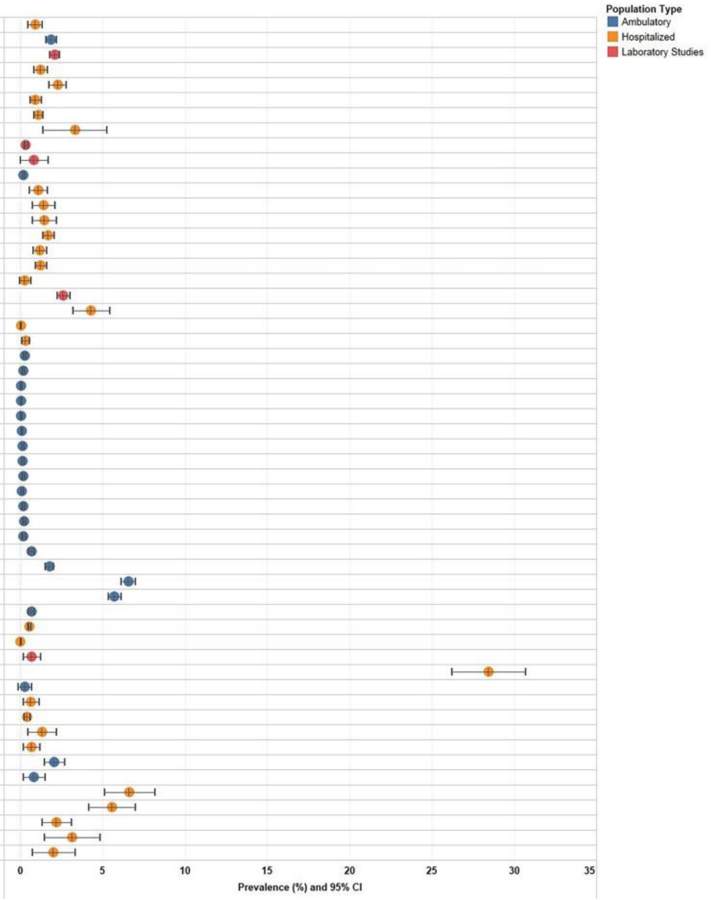

Appendix 5.3 East Asia and Pacific S. Typhi Blood Culture Prevalence

Country	Location	From	To	Author	Year	Sample
Cambodia	Phnom Penh	2006	2009	Kasper	2007	9821
		2007	2010	Vlieghe	2009	445
	Siem Reap	2007	2011	Stoesser	2009	582
Hong Kong	Country-wide	1984	1989	Cheng	1986	371
Indonesia	Badung	2002	2008	Rudiman	2005	1889
Lao PDR	Vientiane	2006	2010	Chansamouth	2008	250
Philippines	Bohol	1994	1996	Herva	1995	1875
	Tagbilaran City	1994	1997	Abucejo	1994	413
					1995	990
					1996	1439
					1997	1857
Thailand	Country-wide	1991	1993	Leelarasamee	1992	1137
		2004	2010	Kanoksil	2007	15251
	Songkhla	2009	2011	Limpitikul	2010	2175
					2011	1611

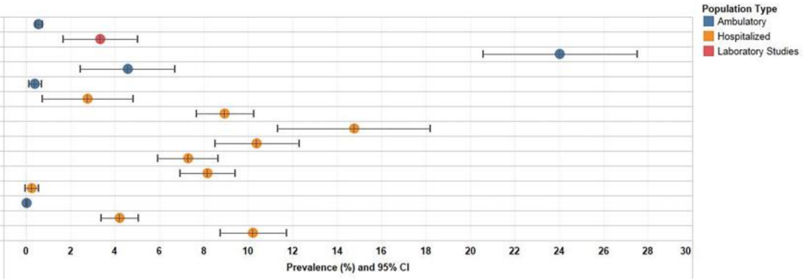

Appendix 5.4 Middle East and North Africa S. Typhi Blood Culture Prevalence

Country	Location	From	To	Author	Year	Sample
Kuwait	Hadiya and Jabriya	1985	1987	Mouaket	1986	221
	Kuwait City	1995	2002	Dimitrov	1995	2359
					1996	2943
					1997	2204
					1998	1729
					1999	1594
					2000	2496
					2001	2789
					2002	2421
Saudi Arabia	Riyadh	1982	1985	Al-Mofleh	1984	62

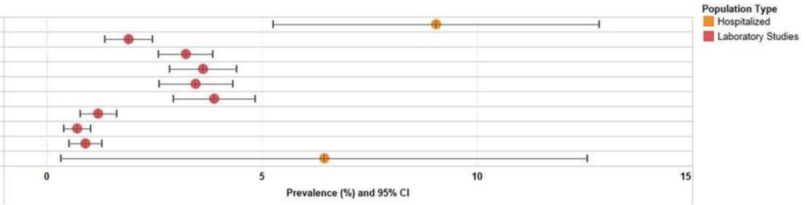

Appendix 5.5 Europe and Central Asia S. Typhi Blood Culture

Country	Location	From	To	Author	Year	Sample
Georgia	Country-wide	2008	2011	Kuchuloria	2010	537
Turkey	Ankara	1993	2002	Ciftci	1998	37

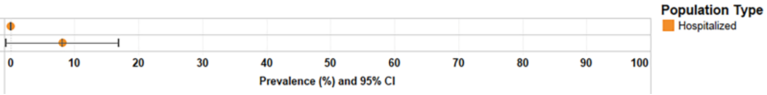

Appendix 5.6 Paratyphoid Blood Culture Prevalence – all regions

Region	Country	Location	From	To	Author	Year	Sample	Organism
East Asia and Pacific	Cambodia	Phnom Penh	2006	2009	Kasper	2007	9821	S. Paratyphi A
			2007	2010	Vlieghe	2009	445	S. Paratyphi A
	Hong Kong	Country-wide	1984	1989	Cheng	1986	371	S. Paratyphi A
								S. Paratyphi B
	Philippines	Tagbilaran City	1994	1997	Abucejo	1996	4699	S. Paratyphi A
Europe and Central Asia	Turkey	Ankara	1993	2002	Ciftci	1998	37	S. Paratyphi B
Middle East and North Africa	Kuwait	Kuwait City	1995	2002	Dimitrov	1995	2359	S. Paratyphi A
						1996	2943	S. Paratyphi A
						1997	2204	S. Paratyphi A
						1998	1729	S. Paratyphi A
						1999	1594	S. Paratyphi A
						2000	2496	S. Paratyphi A
						2001	2789	S. Paratyphi A
						2002	2421	S. Paratyphi A
South Asia	India	Coimbatore	2003	2004	Ravikumar	2003	114	S. Paratyphi A
								S. Paratyphi B
						2004	95	S. Paratyphi A
								S. Paratyphi B
		Ludhiana	2003	2005	Aggarwal	2003	4500	S. Paratyphi A
						2004	5204	S. Paratyphi A
						2005	6571	S. Paratyphi A
		Mangalore	2005	2007	Yashavanth	2007	6297	S. Paratyphi A
		Multiple	1984	1989	Menezes	2007	3745	S. Paratyphi A
		Nagpur	2001	2002	Tankhiwale	2002	452	S. Paratyphi A
		New Delhi	2008	2010	Kharbanda	2009	24091	S. Paratyphi A
				2012	Prasad	2010	2699	S. Paratyphi A
			2010	2012	Jain	2011	13672	S. Paratyphi A
								S. Paratyphi B
		Puducherry	2010	2012	Geetha	2012	14050	S. Paratyphi A
		Rohtak	1997	2001	Gautam	1997	1167	S. Paratyphi A
						1998	1101	S. Paratyphi A
						1999	1162	S. Paratyphi A
						2000	1743	S. Paratyphi A
						2001	1783	S. Paratyphi A
	Nepal	Kathmandu	1993	2003	Maskey	1993	6065	S. Paratyphi A
						1994	6268	S. Paratyphi A
						1995	6643	S. Paratyphi A
						1996	6124	S. Paratyphi A
						1997	5308	S. Paratyphi A
						1998	6025	S. Paratyphi A
						1999	6178	S. Paratyphi A
						2000	5928	S. Paratyphi A
						2001	9059	S. Paratyphi A
						2002	7211	S. Paratyphi A
						2003	17658	S. Paratyphi A
			2002	2004	Blacksell	2003	103	S. Paratyphi A
					Sharma	2003	1774	S. Paratyphi A
			2005	2009	Karkey	2005	14295	S. Paratyphi A
						2006	14018	S. Paratyphi A
						2007	12354	S. Paratyphi A
							54536	S. Paratyphi B
								S. Paratyphi C
						2008	13869	S. Paratyphi A
				2014	Thompson	2009	2118	S. Paratyphi A
			2008	2012	Bajracharya	2008	4474	S. Paratyphi A
						2009	5762	S. Paratyphi A
						2010	7303	S. Paratyphi A
						2011	9466	S. Paratyphi A
						2012	3581	S. Paratyphi A
		Pokhara	2000	2003	Guha	2001	2354	S. Paratyphi A
	Pakistan	Karachi	1987	1991	Hafiz	1990	6153	S. Paratyphi A
								S. Paratyphi B
						1991	6292	S. Paratyphi A
								S. Paratyphi B
			1991	2001	Siddiqui	2000	11668	S. Paratyphi A
			2008	2010	Abdullah	2009	481	S. Paratyphi A
								S. Paratyphi B
		Lahore	2007	2011	Raza	2007	332	S. Paratyphi A
						2008	350	S. Paratyphi A
						2009	309	S. Paratyphi A
						2010	486	S. Paratyphi A
						2011	1055	S. Paratyphi A
		Rawalpindi	1996	2003	Butt	1996	3906	S. Paratyphi A
						1997	3737	S. Paratyphi A
						1998	3670	S. Paratyphi A
						1999	3048	S. Paratyphi A
						2000	3583	S. Paratyphi A
						2001	3866	S. Paratyphi A
						2002	3872	S. Paratyphi A
						2003	3422	S. Paratyphi A
Sub-Saharan Africa	Burkina Faso	Nikoko and Polesgo	2011	2013	Al-Emran	2012	1674	S. Paratyphi A
	Ghana	Accra	2010	2013	Labi	2012	23708	S. Paratyphi A
								S. Paratyphi B
	Guinea-Bissau	Bandim	2011	2013	Al-Emran	2012	1021	S. Paratyphi A
	Kenya	Kibera	2011	2013	Al-Emran	2012	1251	S. Paratyphi A
	Madagascar	Imerintsiatosika and Isotry	2011	2013	Al-Emran	2012	2477	S. Paratyphi A
	Mozambique	Manhiça	2001	2014	Mandomando	2008	41668	S. Paratyphi A
								S. Paratyphi C
	Senegal	Pikine	2011	2013	Al-Emran	2012	1058	S. Paratyphi A
	Tanzania	Moshi	2011	2013	Al-Emran	2012	680	S. Paratyphi A

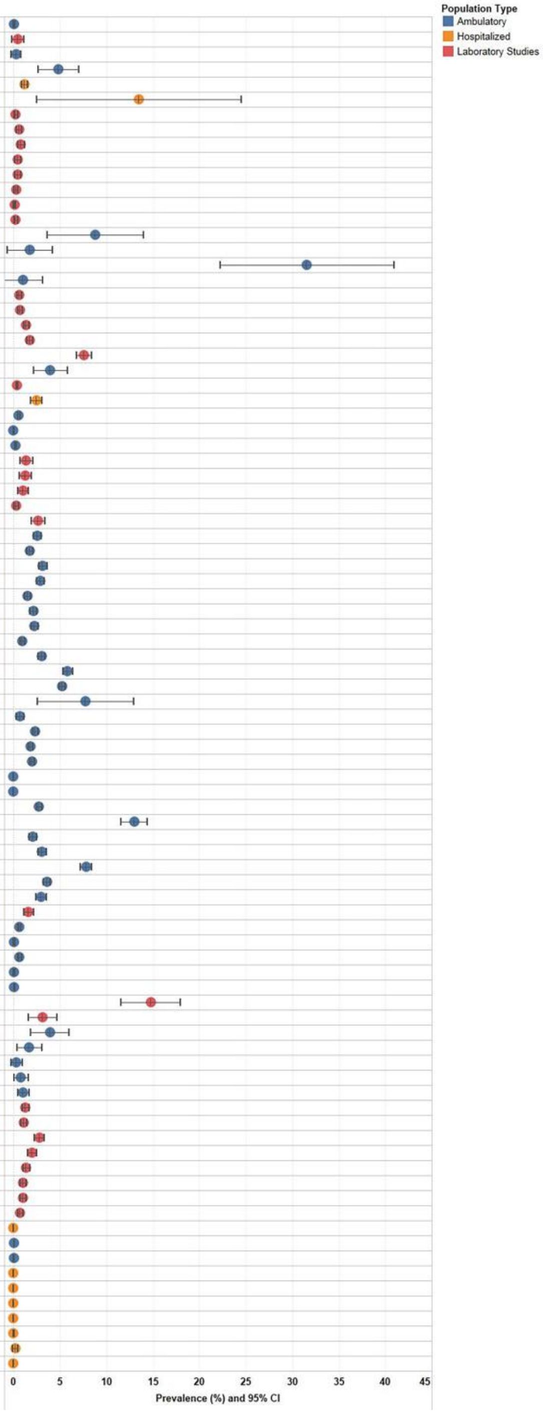

Supplement: Supplementary file 5 [file tpmd180034.SD5.pdf]
