# Supplementary material for: Global Trends in Typhoidal Salmonellosis: A Systematic Review
Source: Am J Trop Med Hyg. 2018 Jul 25;99(3 Suppl):10–9. doi: 10.4269/ajtmh.18-0034 (PMC6128363; doi:10.4269/ajtmh.18-0034)

Appendix 6: Mortality and Morbidity Outcomes

Appendix 6.1 Typhoid ileal perforation morbidity prevalence – all

Region	Country	Location	From	To	Author	Year	Sample
East Asia and Pacific	Thailand	Bangkok	1986	2000	Wongsawat	1993	14
Europe and Central Asia	UK	London	2007	2012	Dave	2010	98
Middle East and North Africa	Kuwait	Kuwait	1985	1989	Daoud	1987	100
South Asia	India	New Delhi	1999	2003	Walia	2001	88
	Nepal	Kathmandu	2009	2011	Singh	2010	138
Sub-Saharan Africa	Nigeria	Maiduguri	1994	2004	Eni	1999	54
	Uganda	Kasese & Bundibugyo	2009	2012	Walters	2010	1042

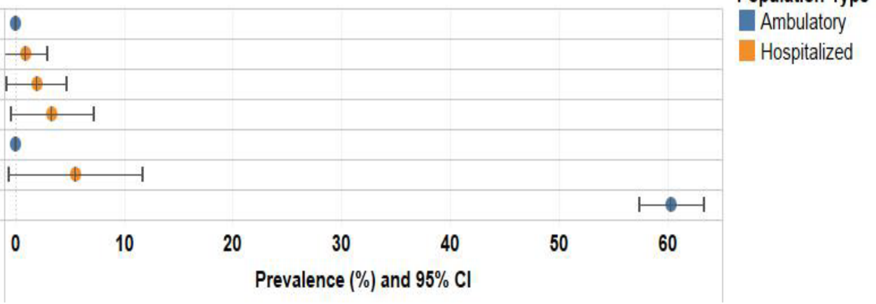

Appendix 6.2 Typhoid ileal perforation case fatality rate – all regions

Region	Country	Location	From	To	Author	Year	Sample
East Asia and Pacific	Thailand	Bangkok	1986	2000	Wongsawat	1993	14
Sub-Saharan Africa	Ivory coast	Abidjan	1995	1998	Kouame	1996	64
	Nigeria	Country-wide	1997	2003	Edino	2000	47
		Enugu	1999	2007	Agu	2003	50
		Ogbomoso	1991	1994	Meier	1992	75

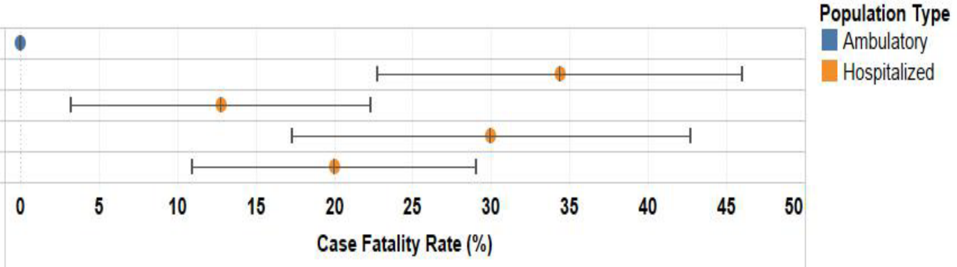

Supplement: Supplementary file 6 [file tpmd180034.SD6.pdf]
